# Supplementary material for: Enhancing extracellular vesicle cargo loading and functional delivery by engineering protein-lipid interactions
Source: Nat Commun. 2024 Jul 4;15:5618. doi: 10.1038/s41467-024-49678-z (PMC11224323; doi:10.1038/s41467-024-49678-z)
Supplement: Supplementary file 1 — Supplementary Information [file 41467_2024_49678_MOESM1_ESM.pdf]

Enhancing extracellular vesicle cargo loading and functional delivery by engineering protein-lipid interactions

*Justin A. Peruzzi**, Taylor F. Gunnels**, Hailey I. Edelstein, Peilong Lu, David Baker, Joshua N. Leonard*, Neha P. Kamat**

*Corresponding authors: j-leonard@northwestern.edu; nkamat@northwestern.edu

**These authors contributed equally.

Contents:

- Figures S1-S23
- Tables S1-S9

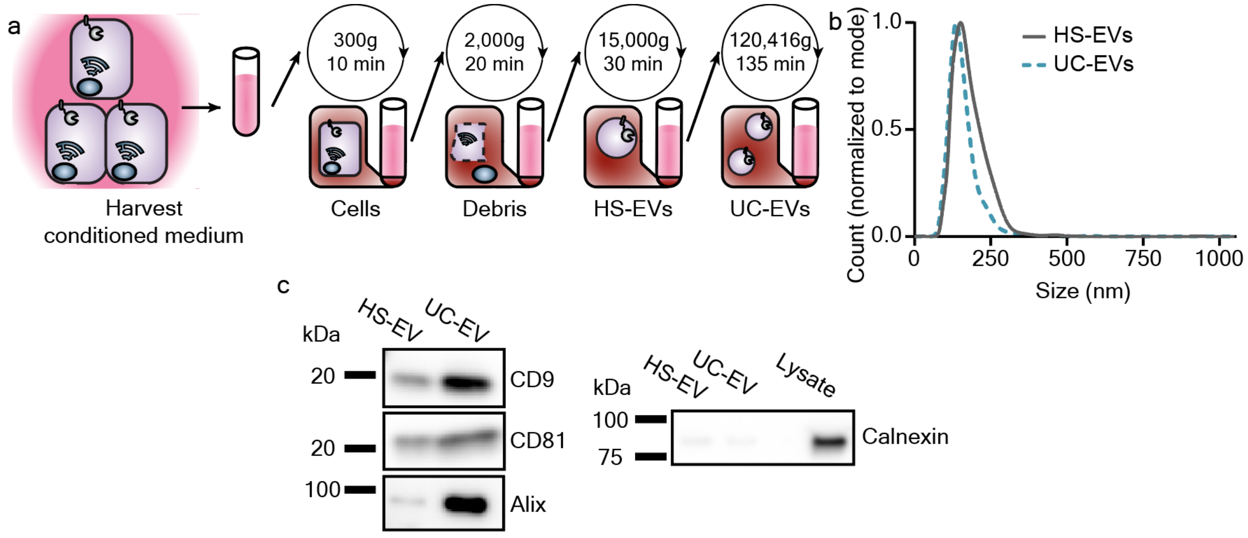

Supplementary Figure 1. Extracellular vesicle (EV) populations from HEK293FT cells demonstrate classical EV characteristics. a Cartoon depicting the process for isolating two EV subtypes, high-speed centrifugation EVs (HS-EVs) and ultracentrifugation EVs (UC-EVs), from conditioned cell culture medium. **b** Representative histogram of particle sizes from the EV subpopulations described in **a** as determined by nanoparticle tracking analysis. **c** Western blots on HEK293FT EV samples and cell lysates for EV markers CD9, CD81, and Alix and the endoplasmic reticulum-associated (non EV-associated) marker, Calnexin (n = 1 independent biological replicate). Equal numbers of EVs were added for each blot. For the Calnexin blot, 4.3 x 10^8 EVs were used per well, and 2.7 µg of lysate was added. Source data are provided as a Source Data file.

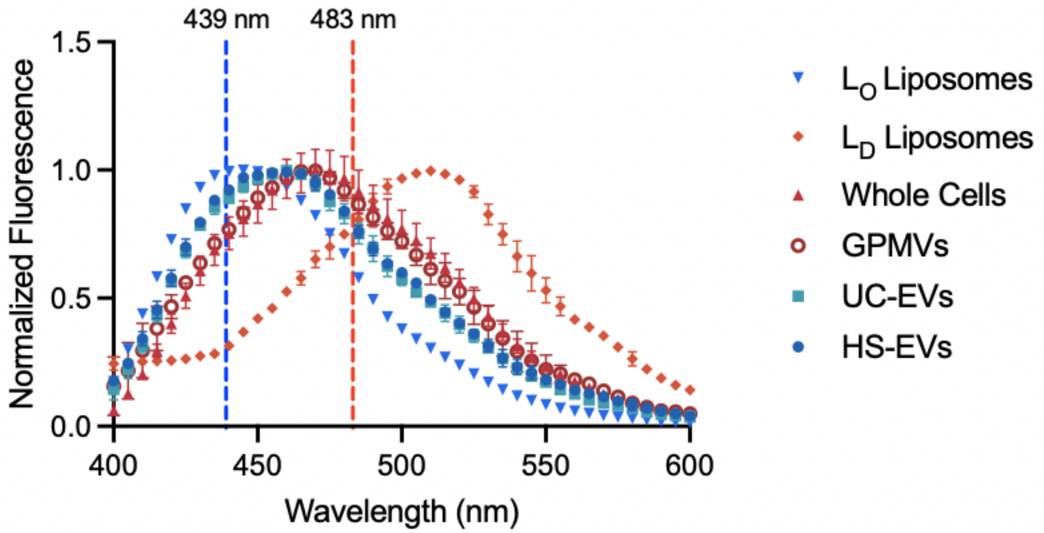

Supplementary Figure 2. Laurdan spectra of samples in Fig. 1c demonstrate that vesicles from the high-speed centrifugation EV fraction (HS-EV) and ultracentrifugation EV fraction (UC-EV) are more similar in lipid order to ordered liposomes (L_O) than disordered liposomes (L_D). L_O liposomes were composed of 70 mol% DPPC/30 mol% Chol, and L_D liposomes were composed of 70 mol% DOPC/30 mol% Chol. Whole cells refers to HEK293FT cells; the EVs in this experiment were derived from HEK293FTs. Spectra were normalized to the maximum fluorescence. Samples were excited by a 350 nm laser and fluorescence intensities were collected from 400 to 600 nm. Intensities at 439 nm (blue line) and 483 nm (red line) were used to calculate Laurdan generalized polarization (GP) via the equation 1 in the methods section. *n*=3 independent biological replicates, error bars represent the SEM. Source data are provided as a Source Data file.

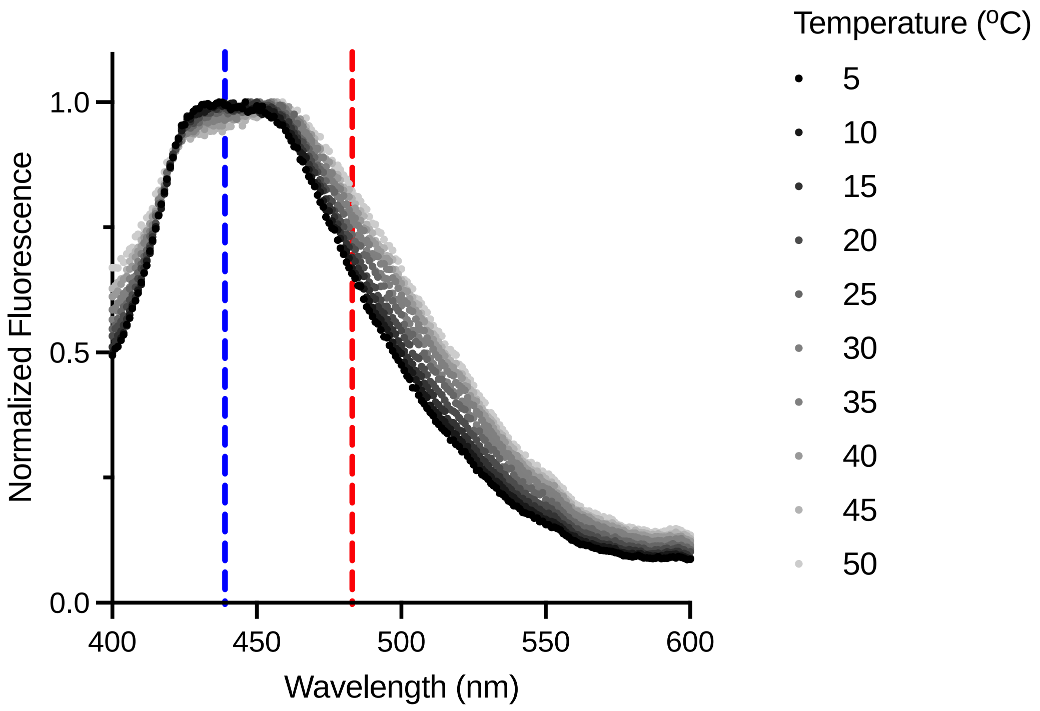

Supplementary Figure 3. Laurdan spectra of UC-EVs as a function of temperature demonstrates a characteristic emission profile. Samples were excited by a 350 nm laser and fluorescence intensities were collected from 400 to 600 nm. Laurdan emission exhibits a slight red shift as temperature increases which reflects the expected decrease in membrane order as temperature increases. Spectra were normalized to the maximum fluorescence. *n*=2 independent sample preparations, error bars represent the SEM. Source data are provided as a Source Data file.

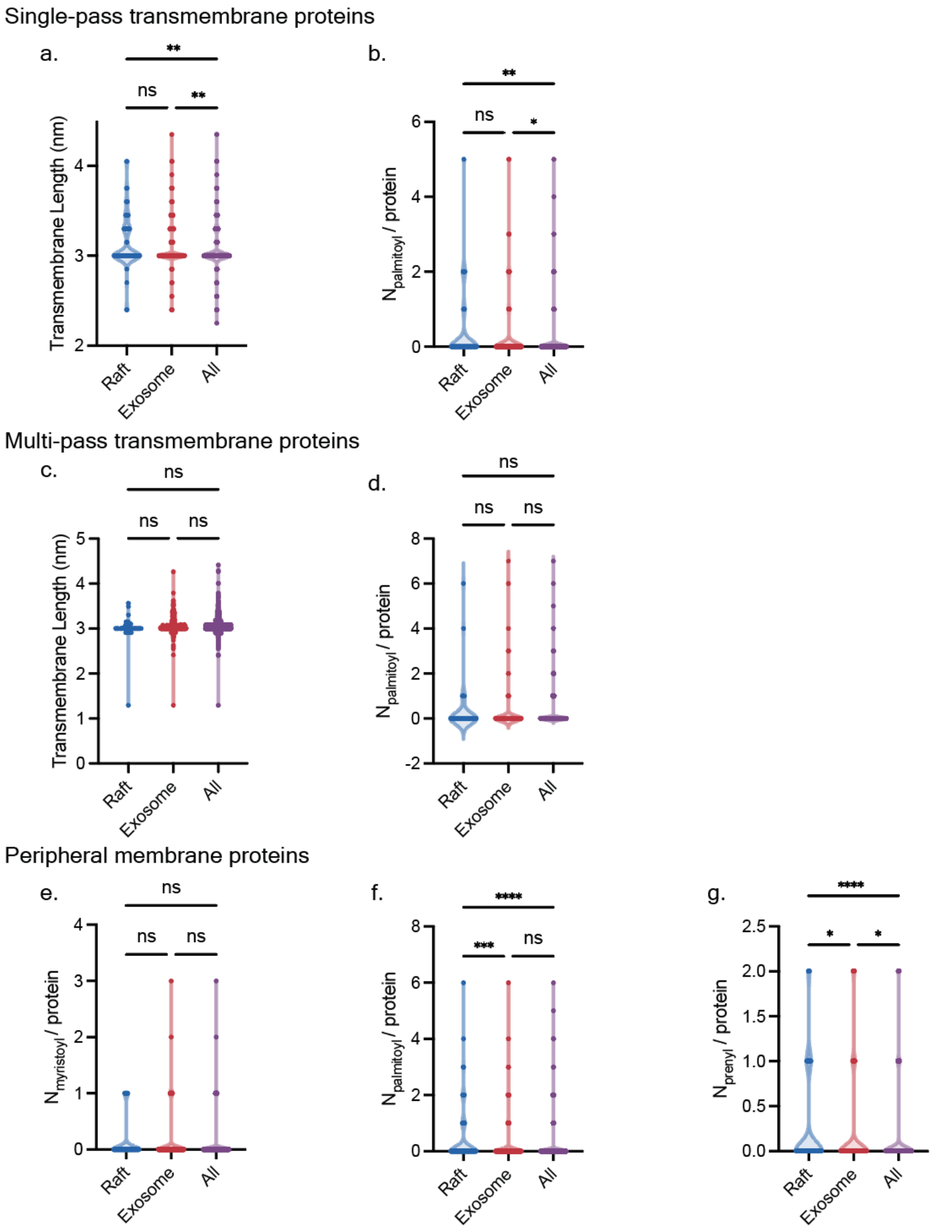

Supplementary Figure 4. Plots of protein structural features presented in Figure 2 as violin plots with individual points. a-g Average transmembrane domain length and number of palmitoyls per protein ($N_{palmitoyl}$/protein) for (**a**, **b**) single-pass and (**c**, **d**) multi-pass transmembrane proteins, and (**e**, **f**, **g**) number of myristoyl, palmitoyl, and prenyl groups on peripheral membrane proteins were compared. Error bars represent SEM, number of proteins in each category can be found in Supplementary Table 1. A Kruskal-Wallis test was performed to compare structural features between each data set, and comparisons were evaluated using the Dunn's multiple comparisons correction (*, $p<0.05$, **, $p<0.01$, ***, $p<0.001$, ****, $p<0.0001$). Source data are provided as a Source Data file.

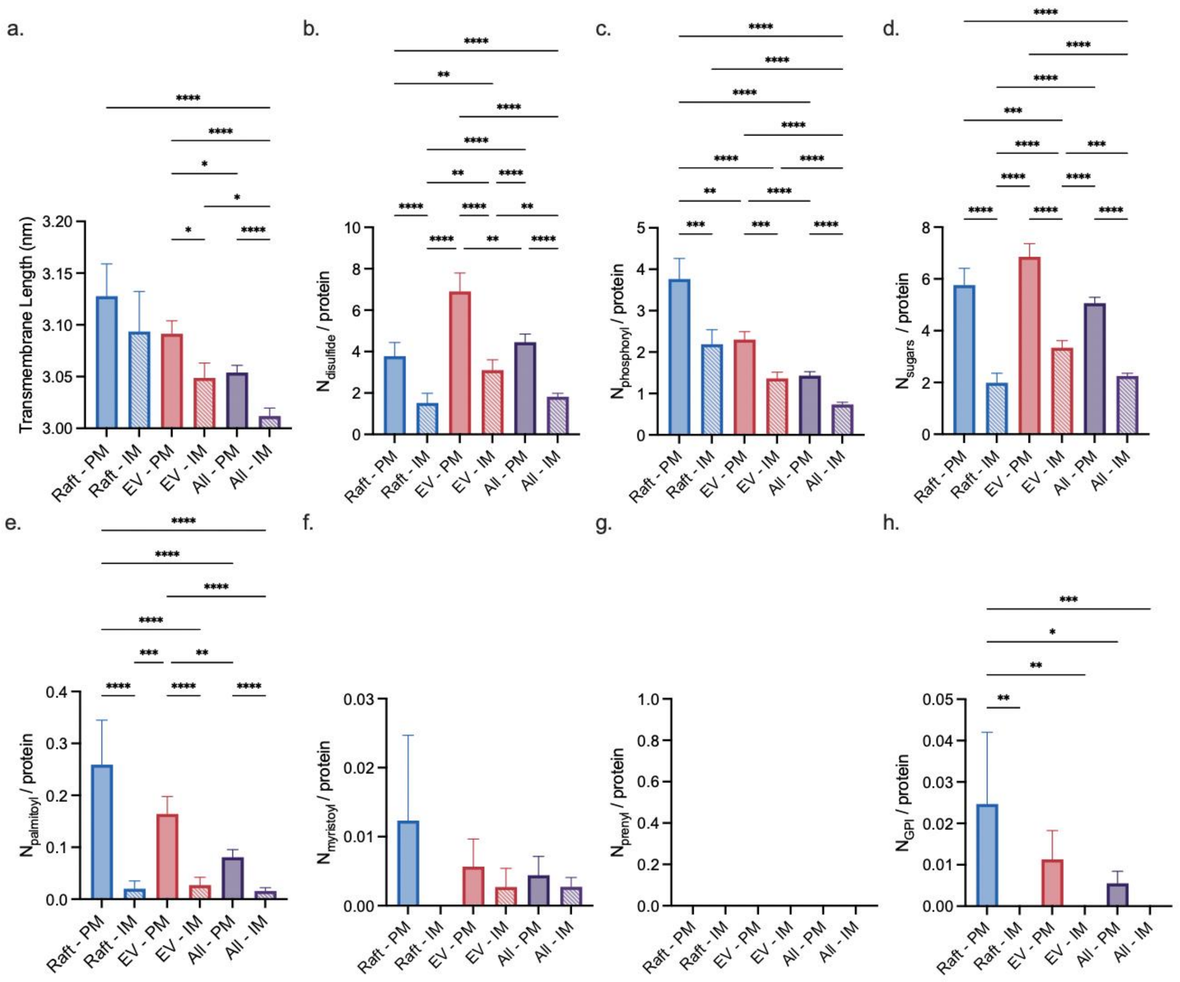

Supplementary Figure 5. For single transmembrane domain proteins, transmembrane domain length and number of posttranslational modifications can vary between proteins found in lipid rafts (Raft, from Raftprot 2.0), EVs (EV, from Exocarta), and all human membrane proteins (All, from Swiss-Prot). Specifically, **a** transmembrane domain length and the average number of **b** disulfides, **c** phosphoryl groups, **d** sugars (glycosylation), **e** palmitoyls, **f** myristoyls, **g** prenyls, and **h** GPI anchors on each protein were calculated. **g** No prenylation of single transmembrane proteins was observed. Proteins were separated by the membrane which they localize to: the plasma membrane (PM) or internal membranes (IM). Error bars represent the SEM, and number of proteins in each category can be found in Supplementary Table 1. A Kruskal-Wallis test was performed to compare structural features between each data set, and comparisons were evaluated using the Dunn's multiple comparisons correction (*, $p<0.05$, **, $p<0.01$, ***, $p<0.001$, ****, $p<0.0001$). Source data are provided as a Source Data file.

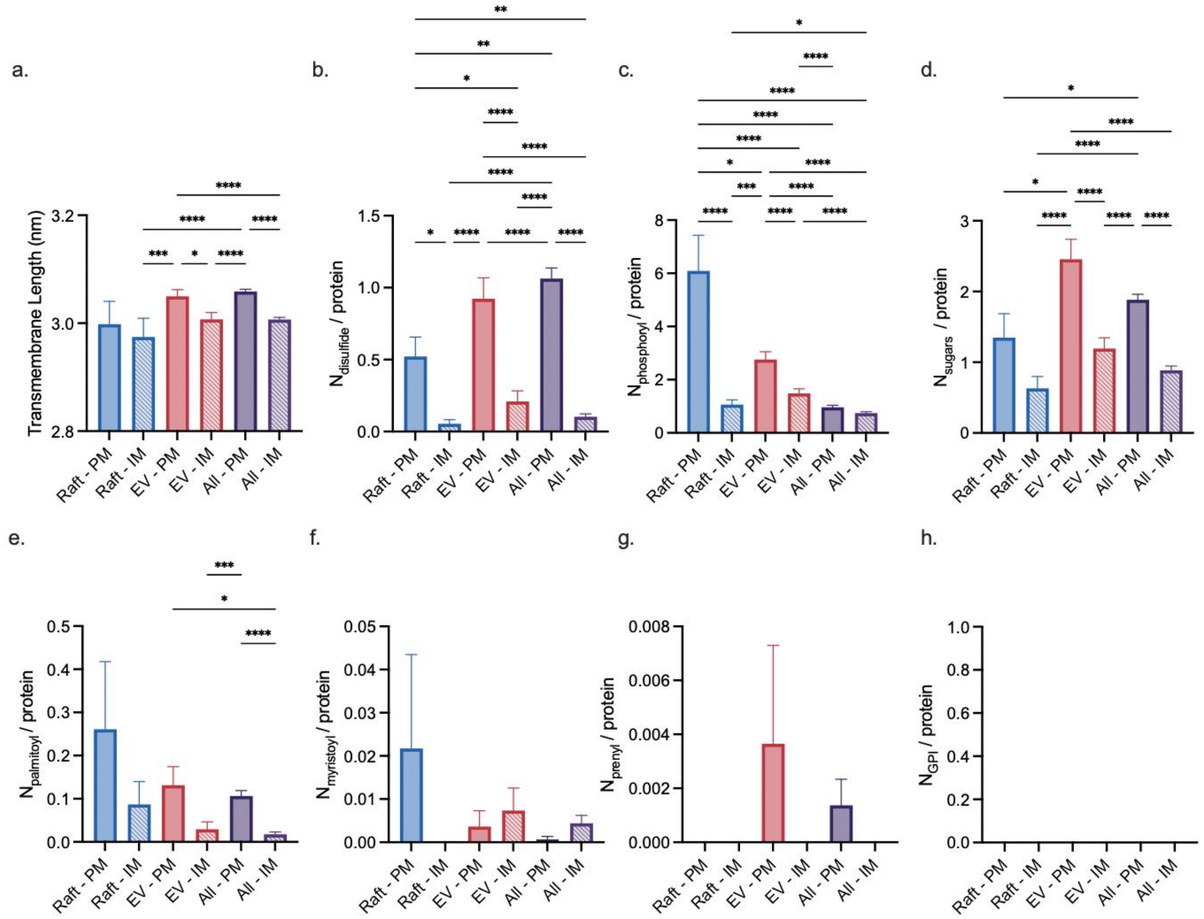

Supplementary Figure 6. For multi-transmembrane domain proteins, transmembrane domain length and number of posttranslational modifications can vary between proteins found in lipid rafts (Raft, from Raftprot 2.0), EVs (EV, from Exocarta), and all human membrane proteins (All, from Swiss-Prot). Specifically, **a** transmembrane domain length and the average number of **b** disulfides, **c** phosphoryl groups, **d** sugars (glycosylation), **e** palmitoyls, **f** myristoyls, **g** prenyls, and **h** GPI anchors on each protein were calculated. **h** No GPI anchors were found on multi-transmembrane proteins. Transmembrane domain length is reported as the average of all transmembrane domains for a single protein. Proteins were separated by the membrane which they localize to: the plasma membrane (PM) or internal membranes (IM). Error bars represent the SEM, and number of proteins in each category can be found in Supplementary Table 1. A Kruskal-Wallis test was performed to compare structural features between each data set, and comparisons were evaluated using the Dunn's multiple comparisons correction (*, $p<0.05$, **, $p<0.01$, ***, $p<0.001$, ****, $p<0.0001$). Source data are provided as a Source Data file.

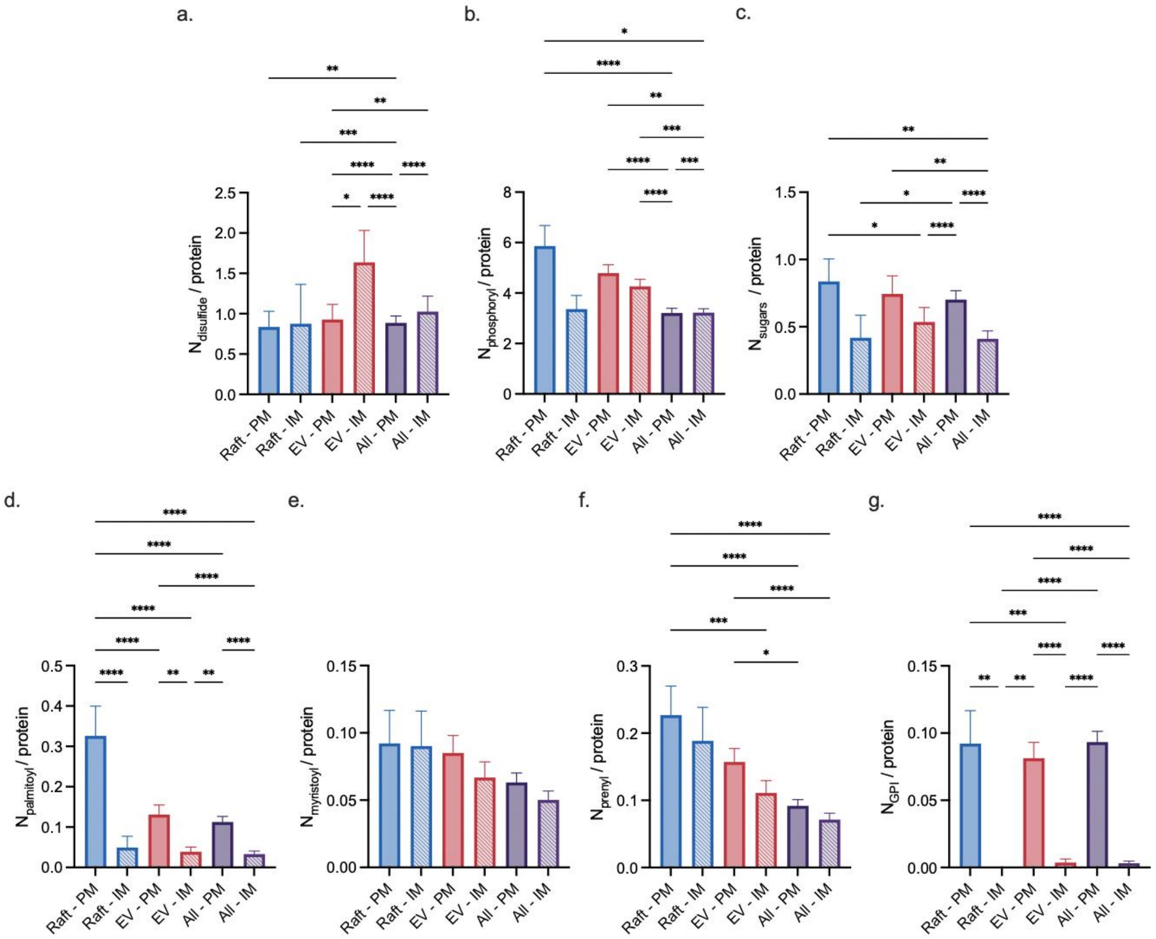

Supplementary Figure 7. For peripheral membrane proteins, number of posttranslational modifications can vary between proteins found in lipid rafts (Raft, from Raftprot 2.0), EVs (EV, from Exocarta), and all human membrane proteins (All, from Swiss-Prot). Specifically, the average number of **a** disulfides, **b** phosphoryl groups, **c** sugars (glycosylation), **d** palmitoyls, **e** myristoyls, **f** prenyls, and **g** GPI anchors on each protein were calculated. Proteins were separated by the membrane which they localize to: the plasma membrane (PM) or internal membranes (IM). Error bars represent the SEM, and number of proteins in each category can be found in Supplementary Table 1. A Kruskal-Wallis test was performed to compare structural features between each data set, and comparisons were evaluated using the Dunn's multiple comparisons correction (*, $p<0.05$, **, $p<0.01$, ***, $p<0.001$, ****, $p<0.0001$). Source data are provided as a Source Data file.

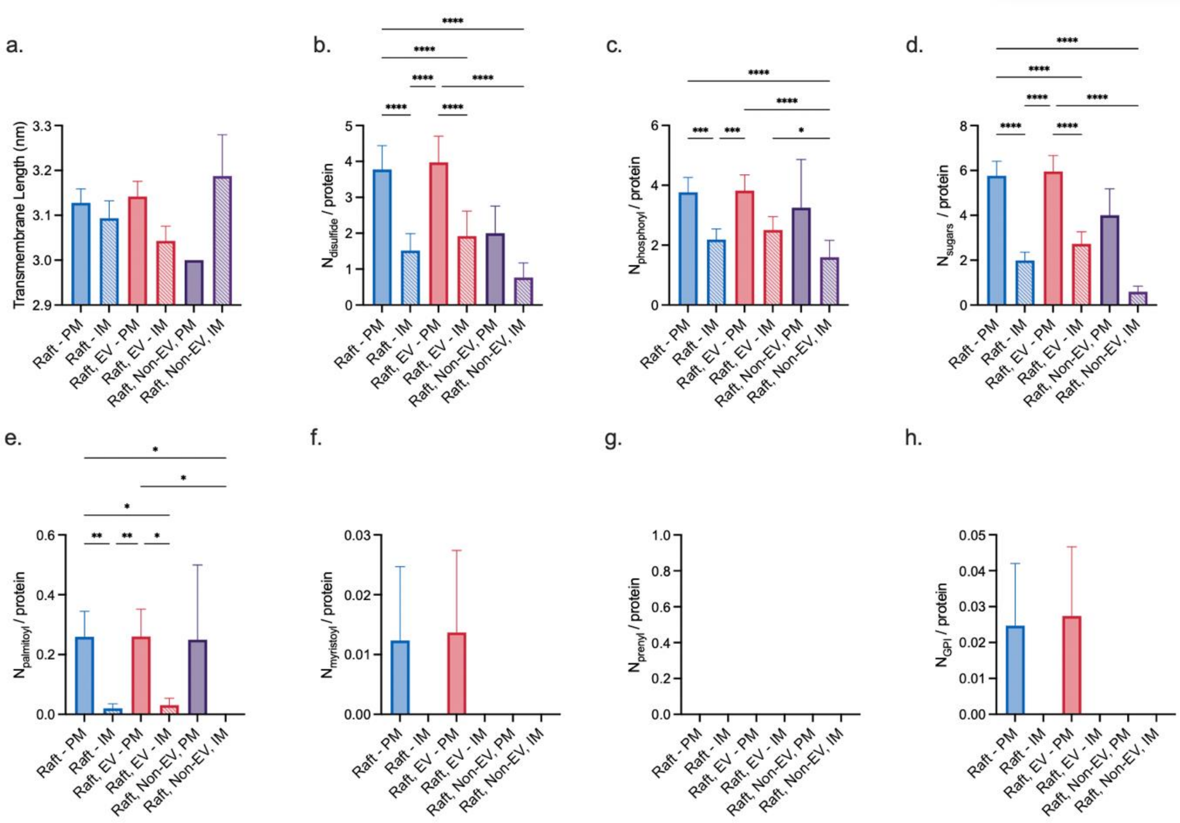

Supplementary Figure 8. For single-pass transmembrane domain proteins, transmembrane domain length and number of posttranslational modifications can vary between proteins found in lipid rafts (Raft, from Raftprot 2.0), rafts proteins found in EVs (Raft, EV, common proteins found in Raftprot 2.0 and Exocarta), and raft proteins not found in EVs (Raft, Non-EV, proteins found in Raftprot 2.0 but not Exocarta). Specifically, **a** transmembrane domain length and the average number of **b** disulfides, **c** phosphoryl groups, **d** sugars (glycosylation), **e** palmitoyls, **f** myristoyls, **g** prenyls, and **h** GPI anchors on each protein were calculated. **g** No prenyl modifications were found on single-pass transmembrane proteins. Proteins were separated by the membrane which they localize to: the plasma membrane (PM) or internal membranes (IM). Error bars represent the SEM, and number of proteins in each category can be found in Supplementary Table 1. A Kruskal-Wallis test was performed to compare structural features between each data set, and comparisons were evaluated using the Dunn's multiple comparisons correction (*, $p<0.05$, **, $p<0.01$, ***, $p<0.001$, ****, $p<0.0001$). Source data are provided as a Source Data file.

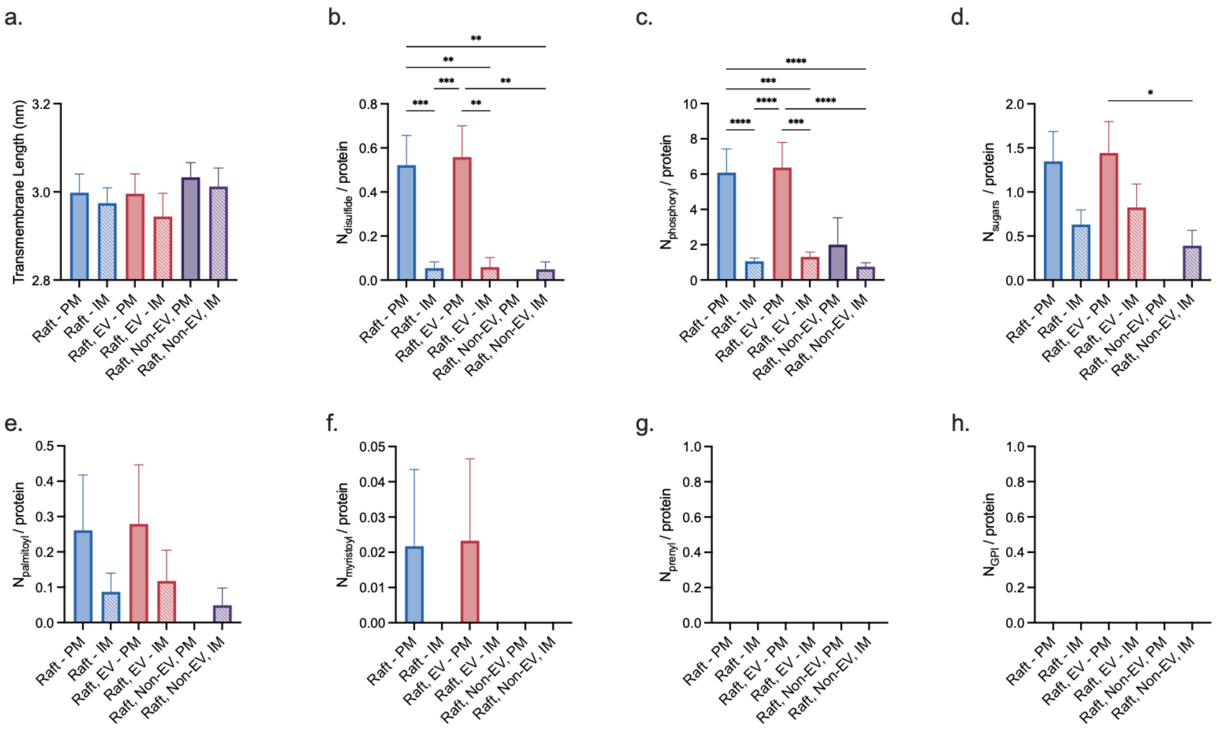

Supplementary Figure 9. For multi-transmembrane domain proteins, transmembrane domain length and number of posttranslational modifications can vary between proteins found in lipid rafts (Raft, from Raftprot 2.0), rafts proteins found in EVs (Raft, EV, common proteins found in Raftprot 2.0 and Exocarta), and raft proteins not found in EVs (Raft, Non-EV, proteins found in Raftprot 2.0 but not Exocarta). Specifically, **a** transmembrane domain length and the average number of **b** disulfides, **c** phosphoryl groups, **d** sugars (glycosylation), **e** palmitoyls, **f** myristoyls, **g** prenyls, and **h** GPI anchors on each protein were calculated. **g**, **h** No prenyl modifications or GPI anchors were found on multi-transmembrane proteins. Transmembrane domain length is reported as the average of all transmembrane domains for a single protein. Proteins were separated by the membrane which they localize to: the plasma membrane (PM) or internal membranes (IM). Error bars represent the SEM, and number of proteins in each category can be found in Supplementary Table 1. A Kruskal-Wallis test was performed to compare structural features between each data set, and comparisons were evaluated using the Dunn's multiple comparisons correction (*, $p<0.05$, **, $p<0.01$, ***, $p<0.001$, ****, $p<0.0001$). Source data are provided as a Source Data file.

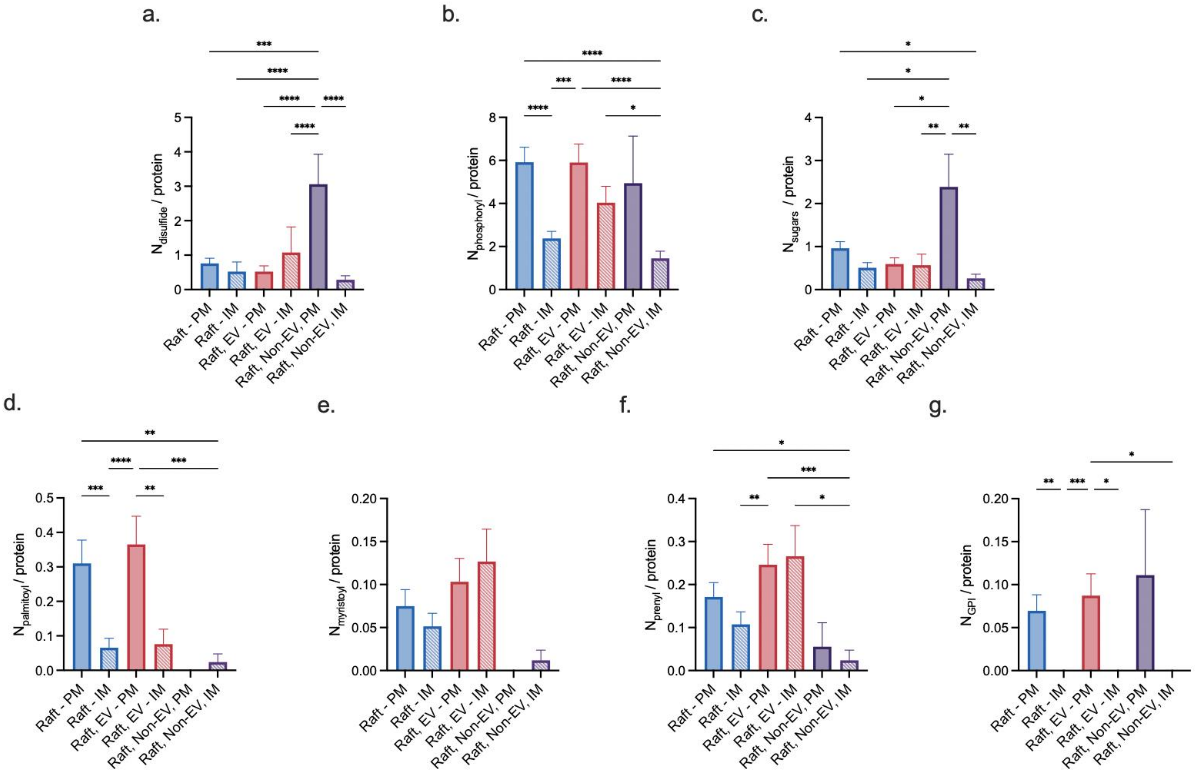

Supplementary Figure 10. For peripheral membrane proteins, the number of posttranslational modifications can vary between proteins found in lipid rafts (Raft, from Raftprot 2.0), rafts proteins found in EVs (Raft, EV, common proteins found in Raftprot 2.0 and Exocarta), and raft proteins not found in EVs (Raft, Non-EV, proteins found in Raftprot 2.0 but not Exocarta). Specifically, the average number of **a** disulfides, **b** phosphoryl groups, **c** sugars (glycosylation), **d** palmitoyls, **e** myristoyls, **f** prenyls, and **g** GPI anchors on each protein were calculated. Proteins were separated by the membrane which they localize to: the plasma membrane (PM) or internal membranes (IM). Error bars represent the SEM, and number of proteins in each category can be found in Supplementary Table 1. A Kruskal-Wallis test was performed to compare structural features between each data set, and comparisons were evaluated using the Dunn's multiple comparisons correction (*, $p<0.05$, **, $p<0.01$, ***, $p<0.001$, ****, $p<0.0001$). Source data are provided as a Source Data file.

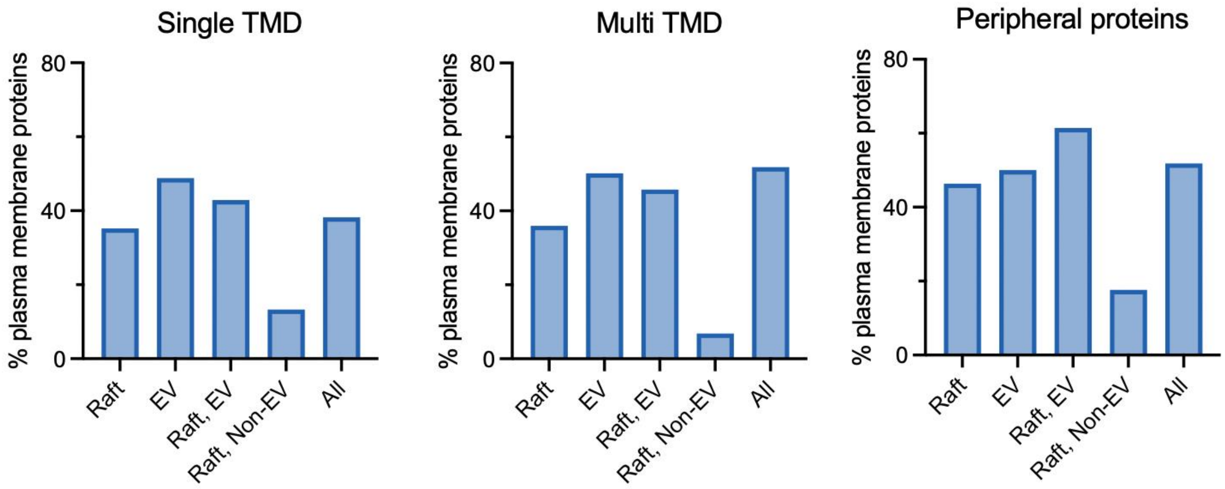

Supplementary Figure 11. The percent of proteins within the following classifications that are found in the plasma membrane for: proteins found in lipid rafts (Raft, from Raftprot 2.0), EVs (EV, from Exocarta), rafts proteins found in EVs (Raft, EV, common proteins found in Raftprot 2.0 and Exocarta), raft proteins not found in EVs (Raft, Non-EV, proteins found in Raftprot 2.0 but not Exocarta), and all human membrane proteins (All, from Swiss-Prot) for single transmembrane domain (TMD) proteins (left), multi transmembrane domain proteins (middle), and peripheral membrane proteins (right). For all three plots, proteins found in rafts but not EVs appear to have the lowest percent of proteins which localize to the plasma membrane. The number of proteins in each category can be found in Supplementary Table 1. Source data are provided as a Source Data file.

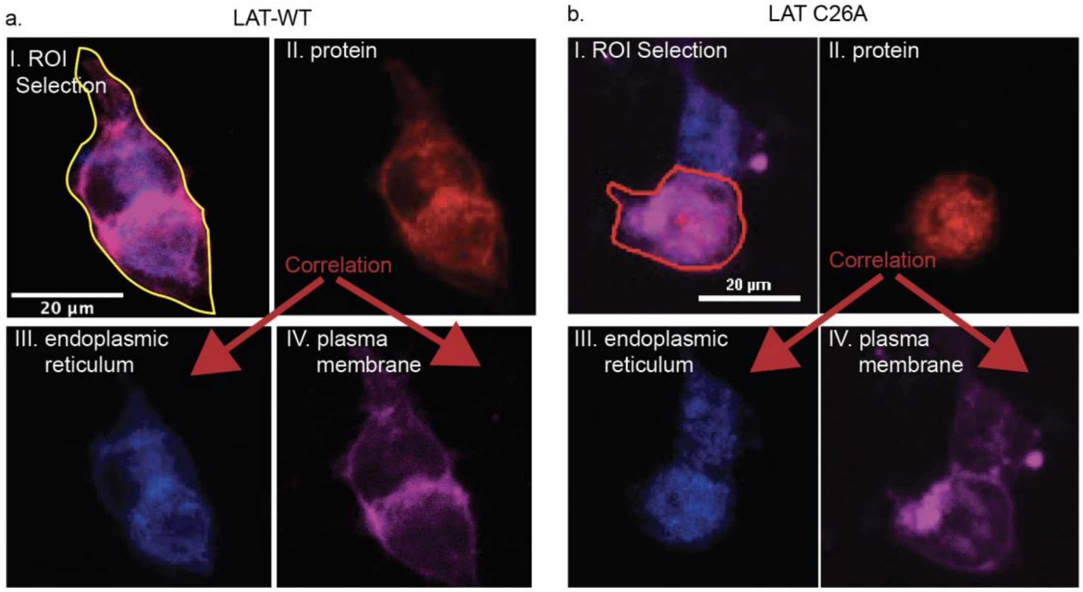

Supplementary Figure 12. Live-cell protein-localization analysis pipeline used in this study enables quantification of protein colocalization with the plasma membrane or endoplasmic reticulum. **a, b** Cells transfected with (**a**) LAT-WT and (**b**) C26A LAT are pictured as an example. HEK293FT cells were transfected with each construct and labeled with (III) ER Tracker Blue-White DPX and (IV) Cell Mask Plasma Membrane Dye. Cells were then imaged (I) and colocalization of protein (II) with each dye (endoplasmic reticulum (III) and plasma membrane (IV)) was determined using Nikon Elements Analysis software.

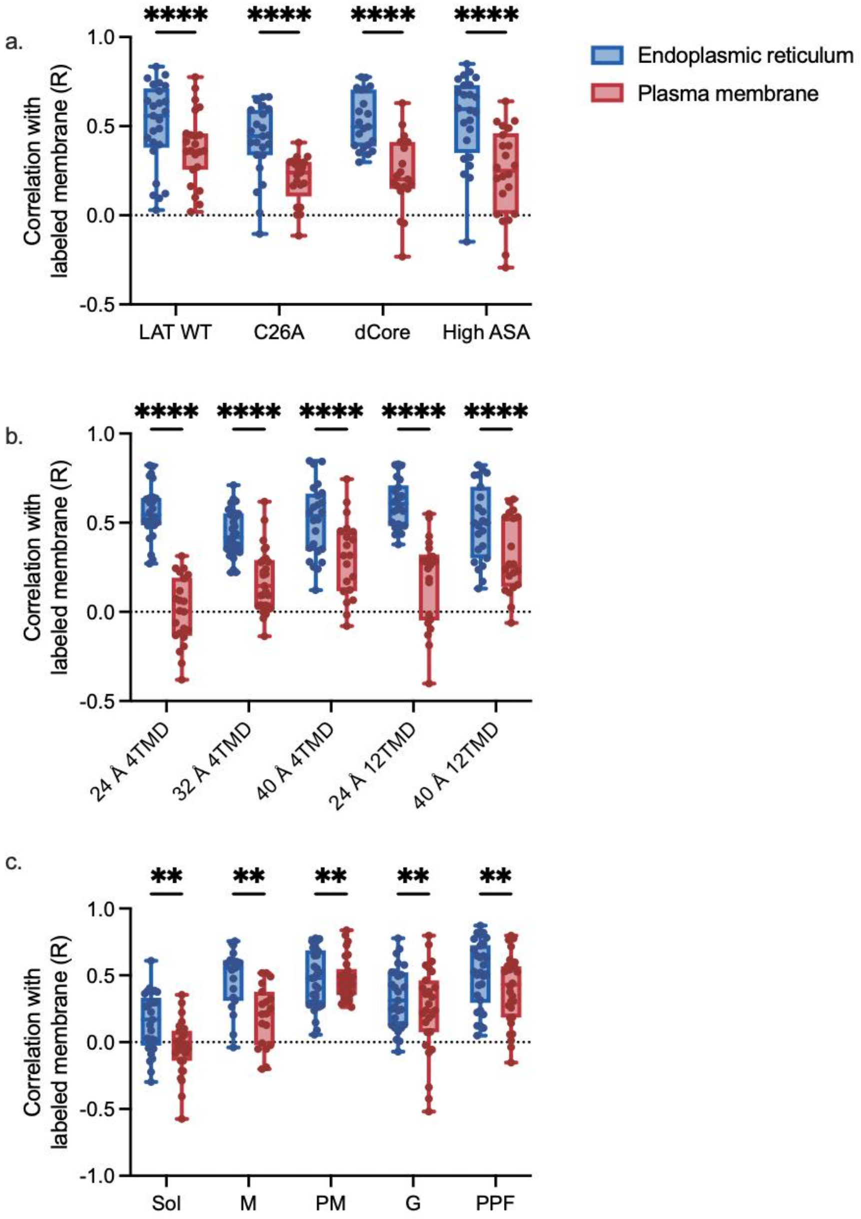

Supplementary Figure 13. Pearson's coefficients (R) between labeled transgenic protein and the plasma membrane and endoplasmic reticulum enable quantification of protein trafficking. a-c The plasma membrane was stained with Cell Mask Plasma Membrane Dye, and the endoplasmic reticulum was labeled with ER Tracker Blue-White DPX dyes. The transfected protein construct was either labeled with HaloTag ligand-conjugated dye (TMR) (**a, c**) or directly visualized by mRFP1 fluorescence (**b**). The data in panel **a**, **b**, and **c** correspond to experiments from Fig. 3, Fig. 4, and Fig. 5, respectively. A two-way ANOVA with main effects only was performed to compare the localization of each protein between the plasma membrane and endoplasmic reticulum, and comparisons were evaluated using a Sidak's multiple comparison test (*, $p<0.05$; **, $p<0.01$; ****, $p<0.0001$). Data are reported in box and whisker plots collected from ≥20 cells from two independent experiments; each symbol is a single cell. The upper and lower bounds represent the minima and maxima of each data measurement, while the box plot

marks the lower and upper quartile, as well as the median. Source data are provided as a Source Data file.

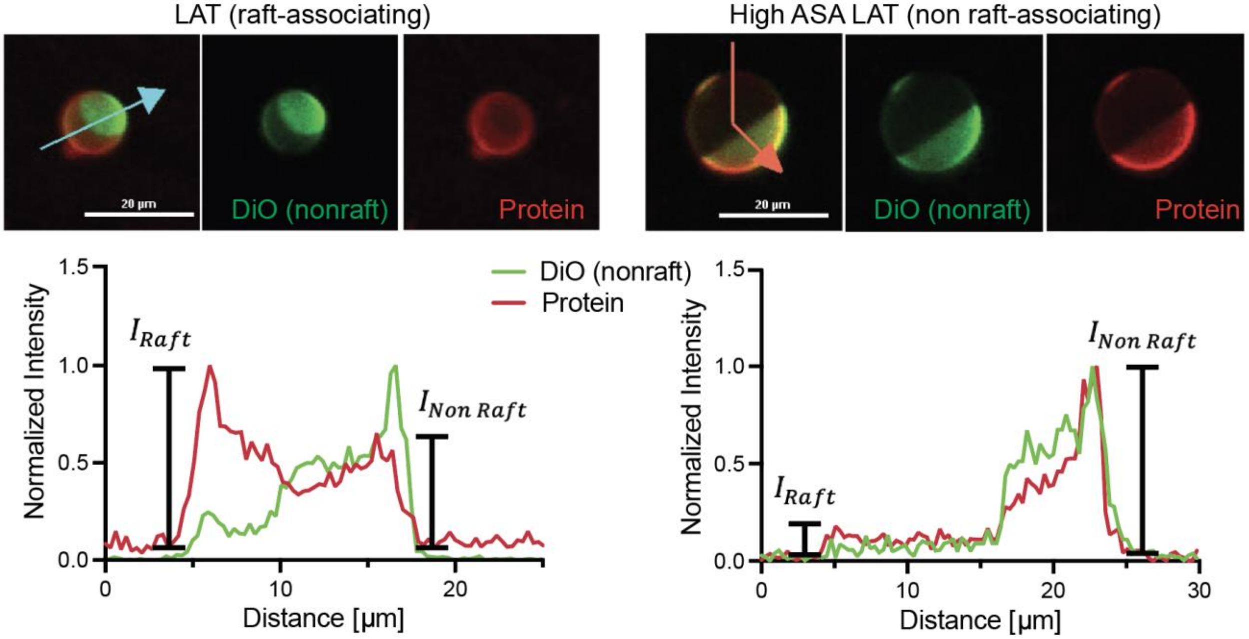

$$L_O\ Enrichment = \frac{I_{Raft} - I_{Non\ Raft}}{I_{Raft} + I_{Non\ Raft}}$$

Supplementary Figure 14. Giant plasma membrane vesicle (GPMV) analysis pipeline enables an evaluation of protein association with lipid rafts. HEK293FT cells were transfected with each construct, treated with vesiculation agents, and stained with DiO (non-raft stain). Protein association with lipid rafts was determined by measuring the protein's fluorescence intensity in the raft region (low DiO fluorescence) and nonraft region (high DiO fluorescence) using line scan analysis and using these values to calculate L_o enrichment (Equation 2). L_o enrichment values above 0 indicate proteins prefer lipid rafts.

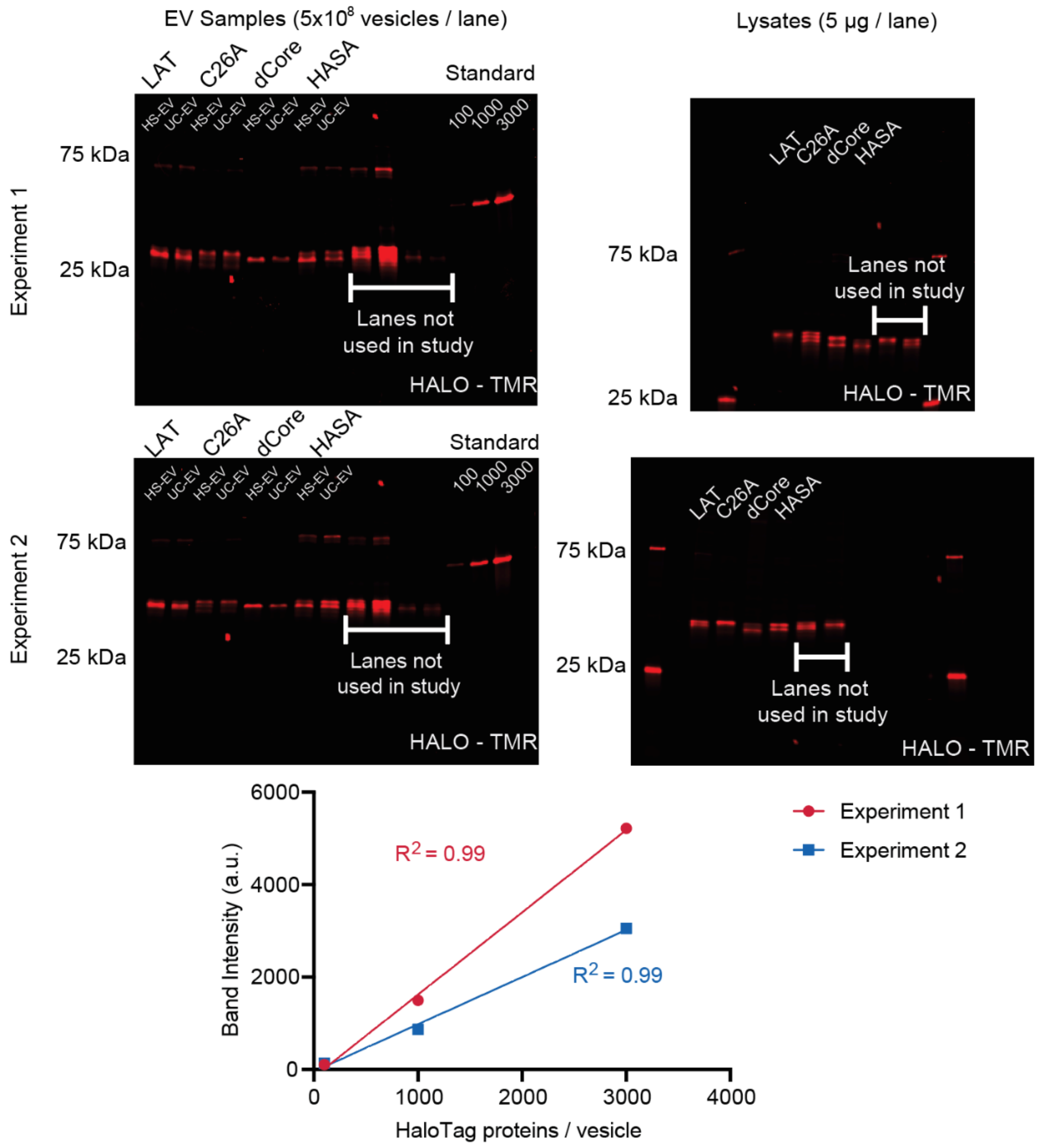

Supplementary Figure 15. Uncropped protein gels and standard curves generated from LAT transmembrane protein gels show construct expression and loading for cell lysates and EVs, respectively. Analyzed data is presented in Fig. 3. Values below the standard indicate the number of recombinant proteins added to each well divided by 5×10^8 (the number of vesicles added to the other wells). Each gel is an independent biological replicate. Source data are provided as a Source Data file.

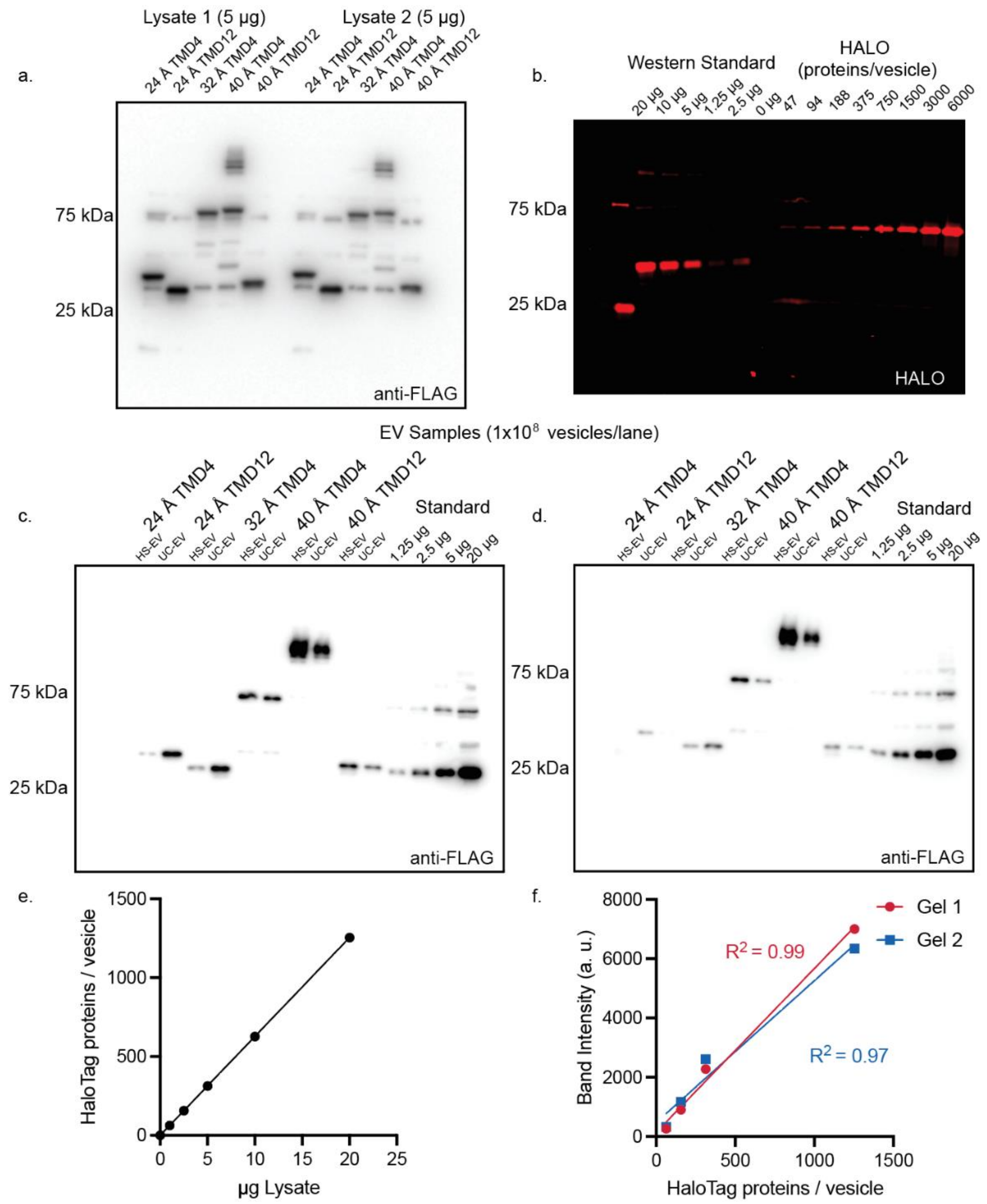

Supplementary Figure 16. Uncropped western blots and standard curves generated from *de novo* designed transmembrane protein gels show construct expression and loading for cell lysates and EVs, respectively. a Uncropped western blot of cell lysates. **b** A protein standard with a 3x FLAG tag and HaloTag was run against purified HaloTag (Promega) on an SDS-PAGE gel to quantify the concentration (and equivalent proteins/vesicle) of the protein standard. **c, d** Proteins loaded into vesicles and protein standards were evaluated via western blot. Constructs labeled with 3x FLAG tag. **e** Protein standard and **f** standard curves used to quantify vesicle loading. Analyzed data are presented in Fig. 4d-e. Each gel is an independent biological replicate. Source data are provided as a Source Data file.

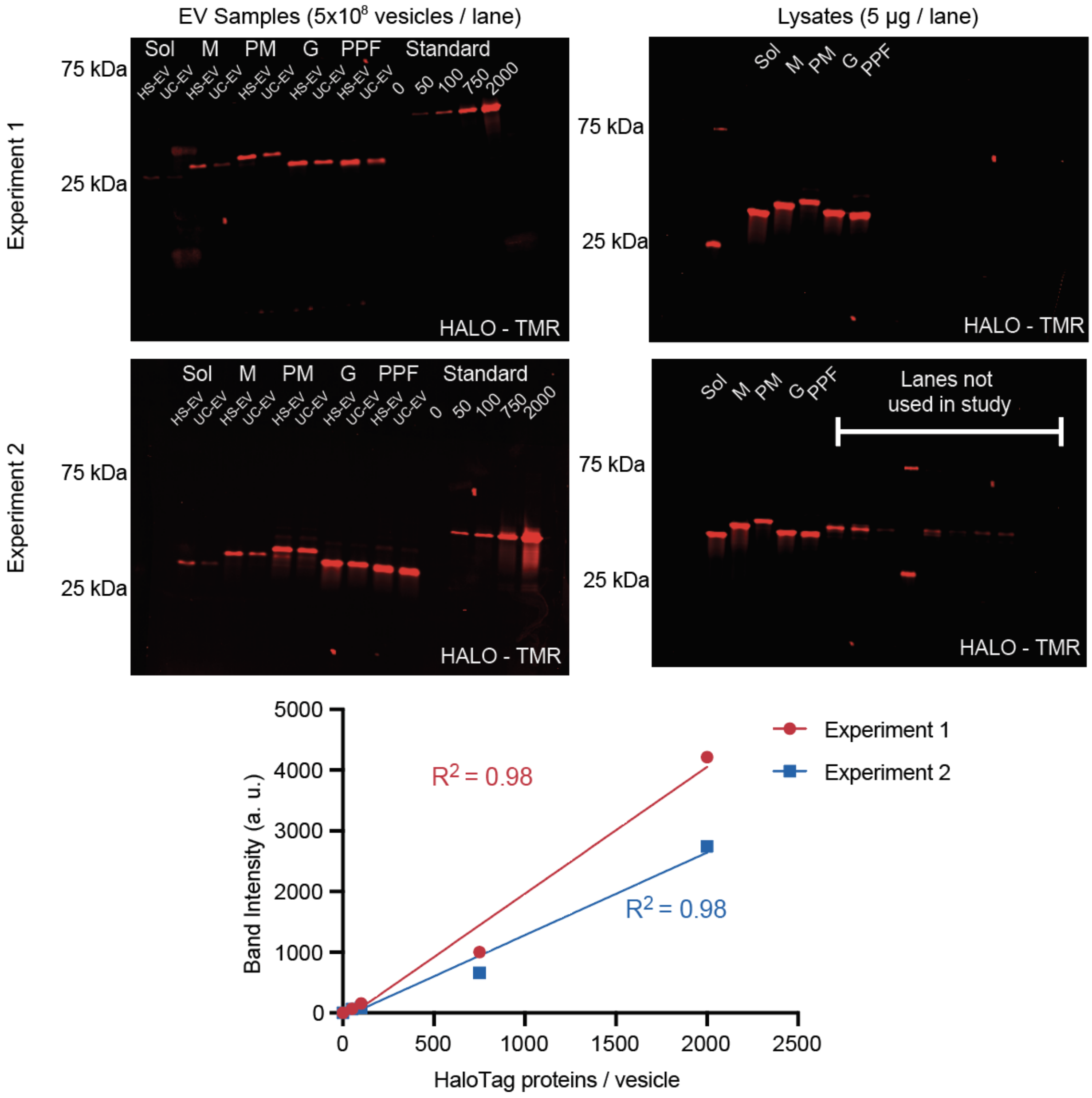

Supplementary Figure 17. Uncropped protein gels and standard curves generated from peripheral membrane proteins show construct expression and loading profiles for cell lysate and EVs, respectively. Analyzed data are presented in Fig. 5. Each gel is an independent biological replicate. Source data are provided as a Source Data file.

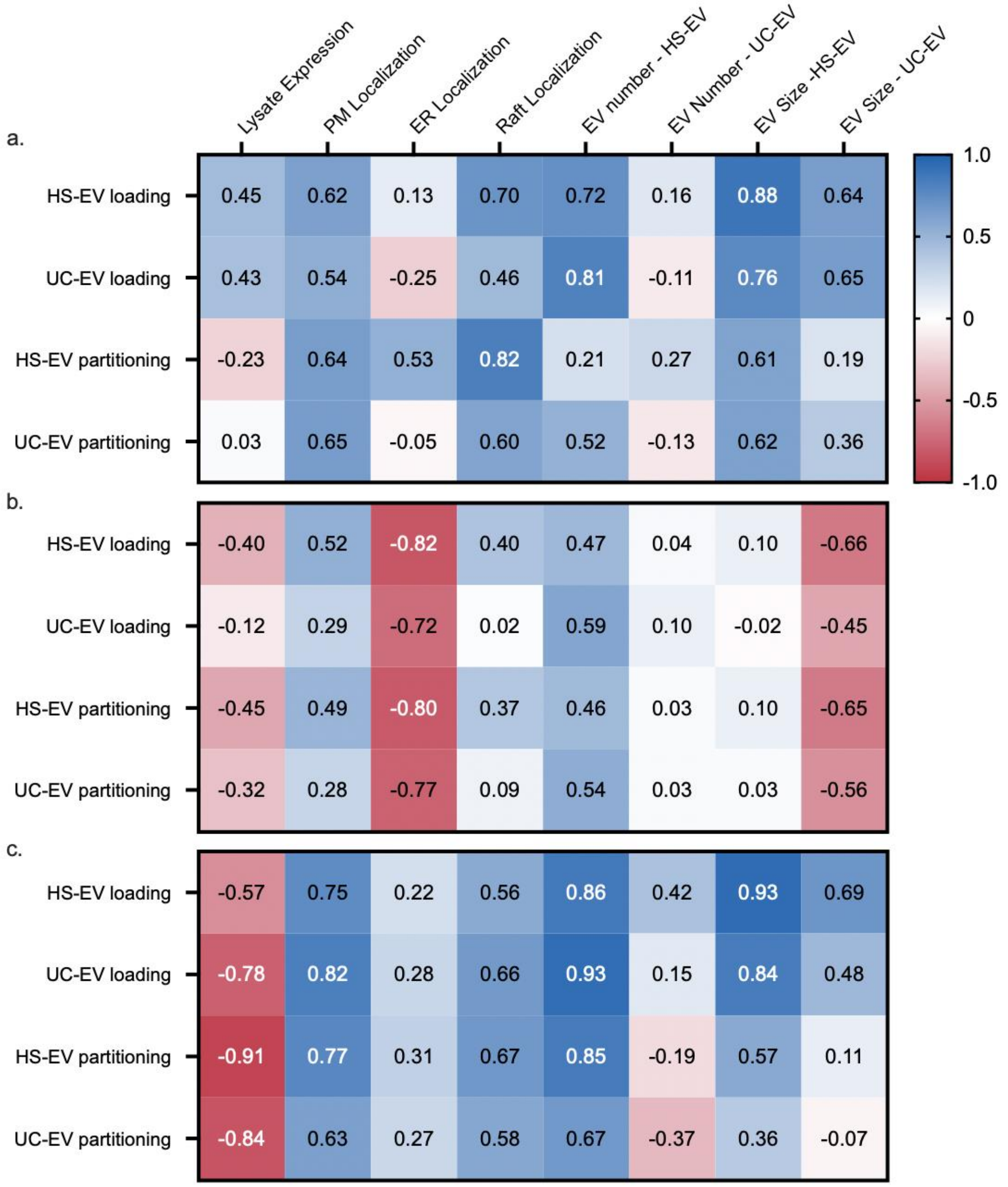

Supplementary Figure 18. Correlation of protein loading and partitioning into HS-EVs and UC-EVs reveal EV-loading design principles. a-c All panels quantify correlation using a Pearson's coefficient. **a** Protein loading and partitioning for LAT proteins (LAT WT, LAT C26A, LAT dCore, LAT High ASA). **b** *de novo* designed proteins (24 Å TMD4, 32 Å TMD4, 40 Å TMD4, 24 Å TMD12, 40 Å TMD12). **c** and lipid tagged HaloTag proteins (Sol, M, PM, G, PPF) were correlated with protein expression in lysate, localization to the plasma membrane and endoplasmic reticulum, association with lipid rafts, and vesicle number and size. Pearson's coefficients and coefficient p-values were calculated by plotting all data against each other using Graph Pad Prism 9. Source data are provided as a Source Data file.

a. Figure 6a- all proteins combined								
	Lysate Expression	PM Localization	ER Localization	Raft Localization	EV number - HS-EV	EV Number - UC-EV	EV Size - HS-EV	EV Size - UC-EV
HS-EV loading	0.6221	0.1908	0.7386	0.0040	0.5096	0.0623	0.9959	0.9239
UC-EV Loading	0.3747	0.2557	0.6723	0.0100	0.3973	0.0114	0.9769	0.5776
HS-EV Partition	0.0004	0.0158	0.7546	0.0130	0.0003	0.2205	0.0021	0.0192
UC-EV Partition	0.0079	0.0242	0.7253	0.0148	0.0066	0.0326	0.0111	0.0348

b. LAT proteins								
	Lysate Expression	PM Localization	ER Localization	Raft Localization	EV number - HS-EV	EV Number - UC-EV	EV Size - HS-EV	EV Size - UC-EV
HS-EV loading	0.2578	0.0993	0.7595	0.0548	0.0425	0.7033	0.0037	0.0876
UC-EV Loading	0.2881	0.1653	0.5476	0.2468	0.0150	0.7884	0.0277	0.0820
HS-EV Partition	0.5779	0.0852	0.1810	0.0122	0.6145	0.5225	0.1089	0.6491
UC-EV Partition	0.9461	0.0828	0.9024	0.1136	0.1877	0.7667	0.0995	0.3802

c. *De novo* designed proteins								
	Lysate Expression	PM Localization	ER Localization	Raft Localization	EV number - HS-EV	EV Number - UC-EV	EV Size - HS-EV	EV Size - UC-EV
HS-EV loading	0.2510	0.1199	0.0038	0.2531	0.1724	0.9145	0.7800	0.0363
UC-EV Loading	0.7478	0.4089	0.0192	0.9528	0.0743	0.7775	0.9456	0.1905
HS-EV Partition	0.1934	0.1471	0.0050	0.2867	0.1789	0.9392	0.7773	0.0398
UC-EV Partition	0.3618	0.4294	0.0097	0.8150	0.1067	0.9244	0.9398	0.0945

d. Lipidated peripheral proteins								
	Lysate Expression	PM Localization	ER Localization	Raft Localization	EV number - HS-EV	EV Number - UC-EV	EV Size - HS-EV	EV Size - UC-EV
HS-EV loading	0.0836	0.0126	0.5396	0.0902	0.0015	0.2312	0.0001	0.0274
UC-EV Loading	0.0079	0.0034	0.4387	0.0365	0.0001	0.6730	0.0023	0.1646
HS-EV Partition	0.0002	0.0086	0.3765	0.0323	0.0021	0.5949	0.0871	0.7554
UC-EV Partition	0.0025	0.0532	0.4493	0.0785	0.0336	0.2947	0.3120	0.8575

Supplementary Figure 19. P-values for correlation data presented in Figure 6a and Supplementary Figure 18. P-values for Pearson's correlation in **a** Figure 6a for all proteins analyzed in this study, **b** LAT proteins (LAT WT, LAT C26A, LAT dCore, LAT High ASA), **c** *de novo* designed proteins (24 Å TMD4, 32 Å TMD4, 40 Å TMD4, 24 Å TMD12, 40 Å TMD12), and **d** lipid tagged HaloTag proteins (Sol, M, PM, G, PPF). Pearson's coefficients and coefficient p-values were calculated by plotting all data against each other using Graph Pad Prism 9. Source data are provided as a Source Data file.

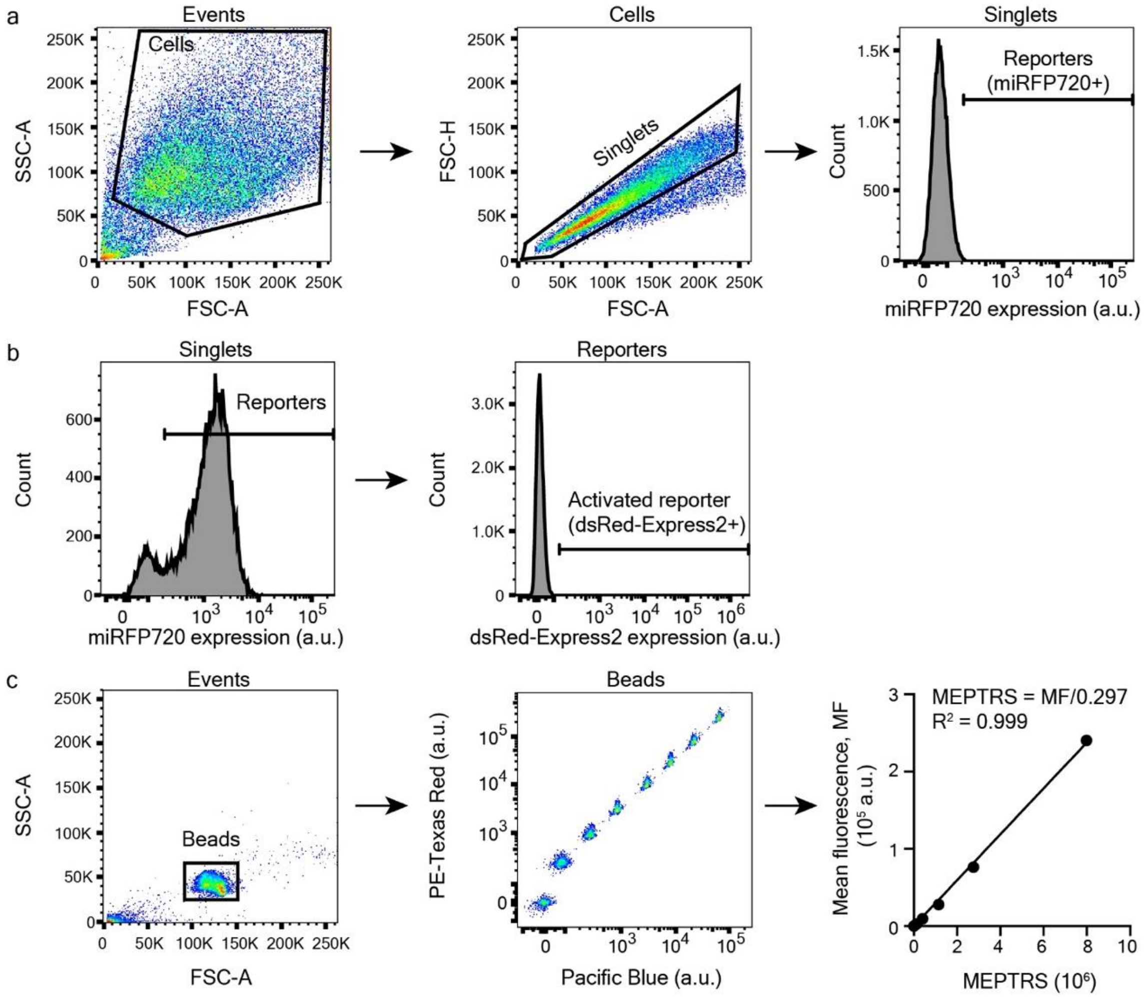

Supplementary Figure 20. Flow cytometry gating and workflow enable quantification of relevant data (e.g. Fig. 7). a Events collected during a typical flow cytometry experiment were first gated based on side scatter area (SSC-A) and forward scatter area (FSC-A) to identify HEK293FT cells. Single cells were then identified by forward scatter height (FSC-H) vs FSC-A discrimination. Single color compensation controls were not gated further prior to generating a compensation matrix. For synTF experiments, HEK293FTs expressing no fluorescent proteins were then used to define the miRFP720+ gate (i.e., reporter cells with an active locus), typically such that < 0.1% of HEK293FTs were considered miRFP720+. **b** Left, a sample histogram of single, reporter cells as gated in **a**. Right, reporter cells that did not receive any synTF were used to establish a dsRed-Express2+ gate. **c** To calibrate fluorescence intensity data, UltraRainbow Calibration Particles were run alongside cell samples for each synTF experiment. Beads were first gated based on SSC-A vs FSC-A (left), and then each bead population was gated using two fluorescent channels, typically PE-Texas Red and Pacific Blue. The mean fluorescence (MF) of each grouping was plotted against the vendor-supplied number of equivalent fluorophores (i.e., molecules of equivalent phycoerythrin-texas red, MEPTRs) and a linear regression was performed with the intercept set to 0. A sample regression equation and goodness of fit are provided.

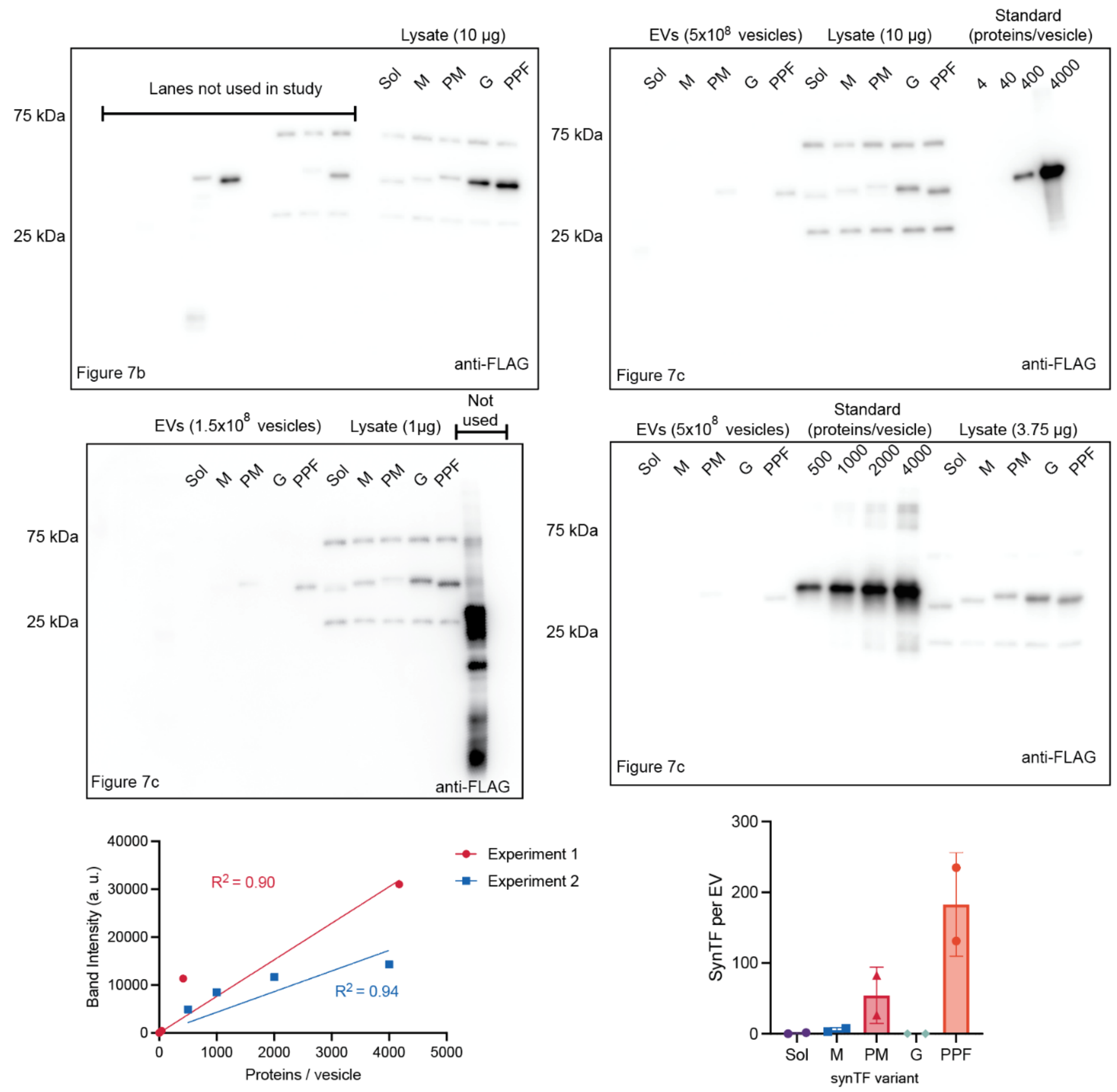

Supplementary Figure 21. Uncropped western blots and standard curves for EV-mediated synTF delivery experiments show construct expression and loading profiles for lysates from EV-producer cells and EVs, respectively. Western blots are probing 1x FLAG tag on the constructs. Protein standard is purified p53 with a 1x FLAG tag (R&D Systems). Analyzed data are presented in Fig. 7b (top left) and 7c (top right and bottom blots). Bottom left, regression curves for the two blots above that were run alongside protein standards. These curves enabled semi-quantitative estimation of the number of synTFs per EV within each blot (bottom right). Band intensities from the western blot were divided by the number of EVs added to the western blot as determined by NTA. All blots are from independent sample preparations and biological replicates (n = 2). Bars represent the mean, the symbols represent an independent sample, and the error bars represent SEM. Source data are provided as a Source Data file.

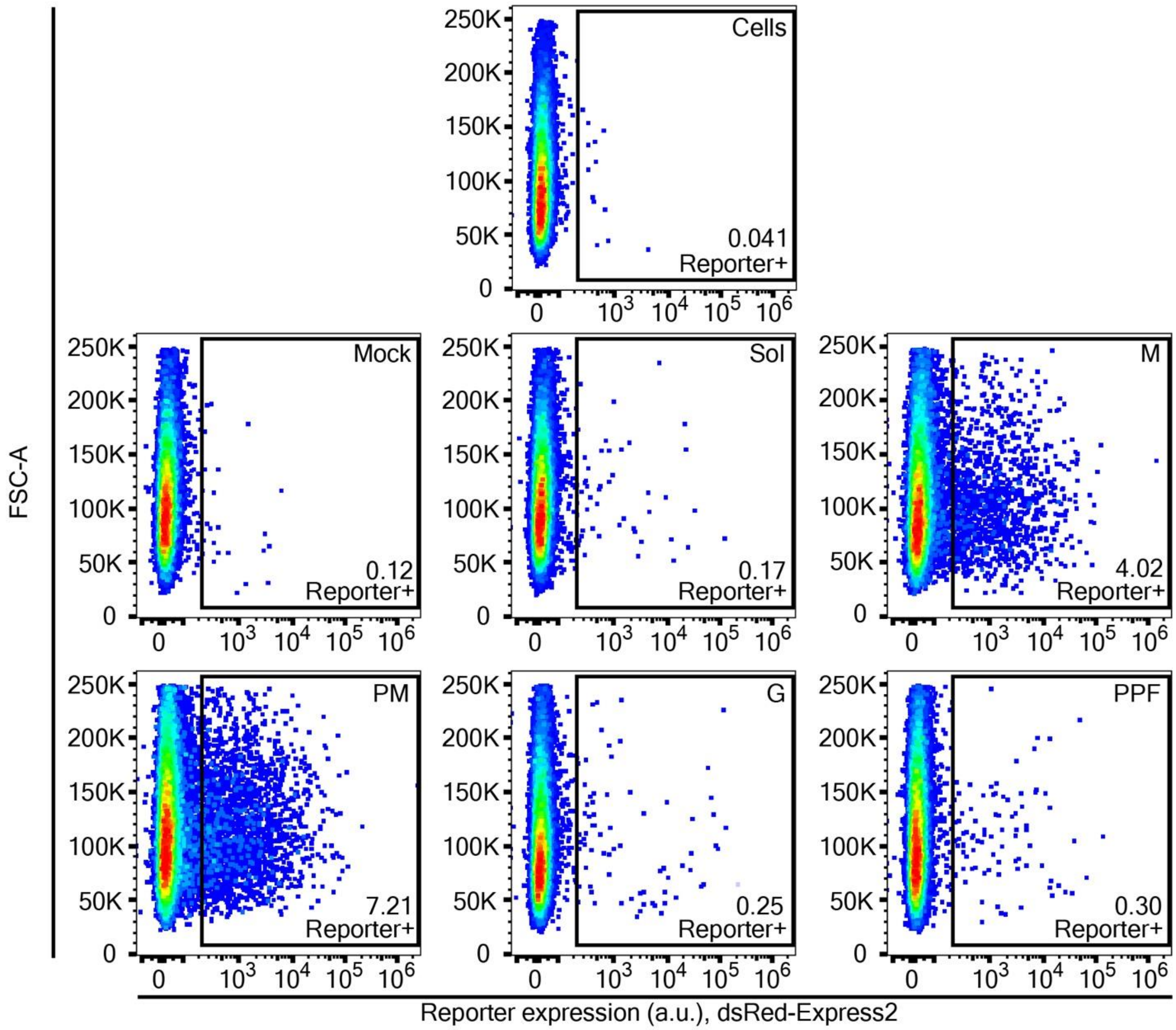

Supplementary Figure 22. Specific lipidated synTF variants induce reporter expression when delivered via EVs. Representative dot plots for each condition from the experiment described in Fig. 7. The plots depict forward scatter area (FSC-A) vs reporter expression (dsRed-Express2) for cells treated with EVs containing lipidated synTFs, where each dot represents an individual reporter cell. The lipidated synTF tag designation is denoted in the upper right-hand corner of each dot plot, and the percent of reporter cells activated is denoted in the lower right hand corner of each dot plot. The Mock and PM conditions are also shown in Fig. 7e.

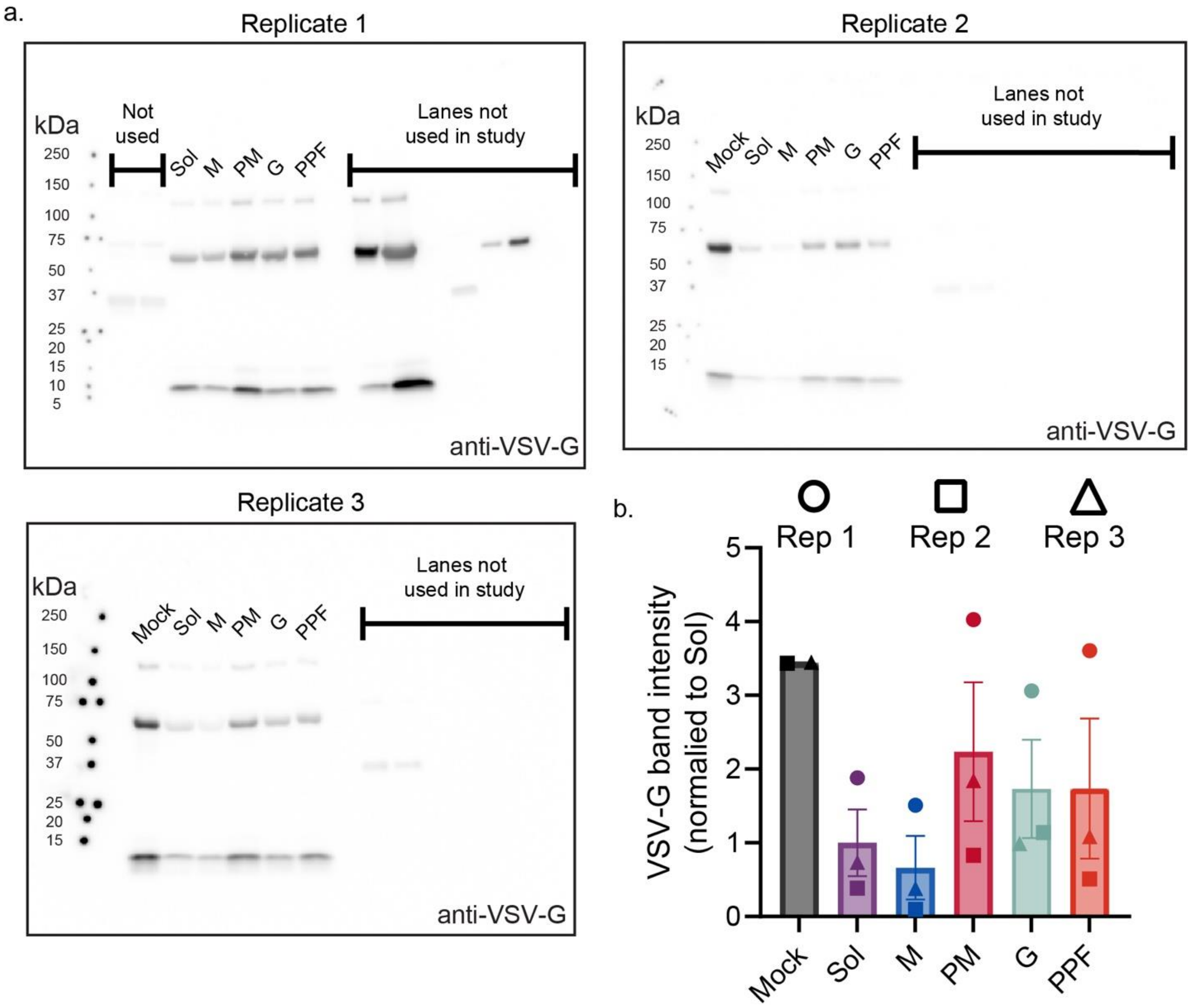

Supplementary Figure 23. Loading of VSV-G in EVs for synTF delivery experiments was similar, but not equal, across SynTF EV preparations. a Raw images of western blots using a primary antibody targeting the C-terminus of the viral fusion protein VSV-G for 3 separate EV preparations (pooled HS-EVs and UC-EVs). The expected size of VSV-G is approximately 60 kDa. The lane titles refer to the synTF plasmid co-transfected into the EV producer cells. $5x10^8$ EVs were loaded per lane. **b** The data in panel **a** were quantified using ImageJ, normalized to the average intensity of the "Sol" across the three preparations, and plotted. The bands near 60 kDa and 120 kDa were analyzed and summed; the band below 15 kDa was presumed to be a fusion-incompetent fragment and was excluded from analysis. Bars represent the average, symbols represent the independent biological replicates (n = 2 or 3), and error bars represent the SEM. Source data are provided as a Source Data file.

Supplementary Table 1. Number of proteins analyzed in bioinformatic study for each protein category.

	Protein	***Single-pass transmembrane protein***	***Multi-pass transmembrane protein***	***Peripheral membrane protein***
Raft	Plasma membrane	81	46	140
	Internal membrane	149	92	163
EV	Plasma membrane	353	274	541
	Internal membrane	370	272	540
EV, raft	Plasma membrane	73	43	122
	Internal membrane	97	51	79
EV, non raft	Plasma membrane	8	3	18
	Internal membrane	52	41	84
All Human Proteins	Plasma membrane	904	1463	1329
	Internal membrane	1463	1360	1235

Supplementary Table 2. Number of EVs produced from HEK293FTs transfected with the below constructs, as determined by nanoparticle tracking analysis*.

Construct	*HS-EVs produced*	*HS-EV Std*	*UC-EVs produced*	*UC-EV Std*
Sol HaloTag	8.26E+09	1.28E+09	1.02E+10	1.56E+09
M HaloTag	9.32E+09	1.41E+09	1.08E+10	1.66E+09
PM HaloTag	1.67E+10	2.52E+09	1.21E+10	1.86E+09
G HaloTag	1.40E+10	2.14E+09	1.34E+10	2.10E+09
PPF HaloTag	1.53E+10	2.34E+09	7.04E+09	1.24E+09
LAT WT	6.97E+09	1.12E+09	8.16E+09	1.27E+09
LAT C26A	5.98E+09	9.43E+08	6.90E+09	1.10E+09
LAT dCore	5.87E+09	8.91E+08	8.74E+09	1.37E+09
LAT High ASA	8.01E+09	1.22E+09	8.02E+09	1.26E+09
24 Å 4TM	5.91E+09	8.96E+08	1.04E+10	1.60E+09
32 Å 4TM	7.06E+09	1.07E+09	1.14E+10	1.76E+09
40 Å 4TM	6.95E+09	1.07E+09	1.23E+10	1.85E+09
24 Å 12TM	7.09E+09	1.10E+09	8.37E+09	1.28E+09
40 Å 12TM	5.54E+09	8.54E+08	1.24E+10	1.89E+09

* n=2

Supplementary Table 3. EV size for vesicles harvested from HEK293FTs that were transfected with the below constructs, as determined by nanoparticle tracking analysis*.

	HS-EV Size (nm)	***HS-EV Std (nm)***	***UC-EV Size (nm)***	***UC-EV Std (nm)***
Sol HaloTag	197.4	72.9	160.1	45.9
M HaloTag	202.7	77.9	165.7	47.5
PM HaloTag	205.7	71.3	164.5	51.6
G HaloTag	207.6	70.2	171.3	50.7
PPF HaloTag	203.5	71.4	161.9	57.0
LAT WT	191.0	59.6	160.3	50.5
LAT C26A	184.6	68.0	158.6	51.2
LAT dCore	185.8	71.6	159.6	50.1
LAT High ASA	190.3	72.8	161.8	49.9
24 Å 4TM	173.8	64.3	151.3	51.5
32 Å 4TM	174.8	64.5	148.3	46.3
40 Å 4TM	172.4	57.3	155.2	46.8
24 Å 12TM	181.6	65.1	148.9	51.4
40 Å 12TM	173.0	61.2	153.9	45.7

*n=2

Supplementary Table 4. Protein source and sequences for proteins used in this study.

TAG	PROTEIN SOURCE	PROTEIN SEQUENCE
**LAT WT**	LAT	MEEAILVPCVLGLLLLPILAMLMALCVHCHRLP - [POI]
**LAT C26A**	LAT-modified	MEEAILVPCVLGLLLLPILAMLMALAVHCHRLP - [POI]
**LAT DCORE**	LAT-modified	MEEAILVPCVLGLAMLMALCVHCHRLP - [POI]
**LAT HIGH ASA**	LAT-modified	MEEAILVLLLLLLLLLPLLLLLLLLCVHCHRLP - [POI]
**24 Å 4TMD**	*de novo* designed	MGSTRTEIIRELERSLREQRVLAIFLLALLIVLLWLLQQLKELLRELERLQREGSSDEDVRELLREIKELVENIVYLVIIIMVLVLVIIALARTQKYLVEELKRQD - [POI]
**32 Å 4TMD**	*de novo* designed	MGSTRTEIITRLSFSLLLQLVLAIFLLALLIVLLWLLQQLKELLRELERLQREGSSDEDVRELLREIKELVENIVYLVIIIMVLVLVIIALAVLQMYLVRELKRQD - [POI]
**40 Å 4TMD**	*de novo* designed	MGSTRTEIITRLSFSLLLQLVLAIFLLALLIVLLVLLIYLKELLRELERLQREGSSDEDVRELLREIKWLVIVIVALVIIIMVLVLVIIALAVLQMYLVRELKRQD - [POI]
**24 Å 12TMD**	*de novo* designed	MTENEIRKLRKLLRIAMFLLVFLLIWTWISLETSKTDDDPSAQSEALVAMSLMLIAASLLIIAKSKLMKSRNG - [POI]
**40 Å 12TMD**	*de novo* designed	MTKKIIMVLILLLIIAMLLLVFLLIATVVSLWWSWTDDDPSAISEALVAMSLMLIAASLLIIAISKLLKSKNG - [POI]
**M**	Src	MGSSKSKPKDPSQRRNNNNGPVAT - [POI]
**PM**	Lyn	MGCIKSKRKDKDLELKLRILQSTVPRARDPPVAT - [POI]
**G**	K-Ras	[POI]-FRSDGKKKKKKSKTKCQLL
**PPF**	Paralemmin-1	[POI]-CKCCSIM

Supplementary Table 5. DNA sequences for protein loading studies.

Construct	DNA Sequence
**WT LAT HaloTag***	ATGGAGGAGGCCATCCTGGTCCCCTGCGTGCTGGGGCTCCTGCTGCTGCCCATCCTGGCCATGTTGAT<br>GGCACTGTGTGTGCACTGCCACAGACTGCCAGGCTCCGGATCCGGCGGCTCCGAAATCGGTACTGGCT<br>TTCCATTCGACCCCCATTATGTGGAAGTCCTGGGCGAGCGCATGCACTACGTCGATGTTGGTCCGCGCG<br>ATGGCACCCCTGTGCTGTTCCTGCACGGTAACCCGACCTCCTCCTACGTGTGGCGCAACATCATCCCGC<br>ATGTTGCACCGACCCATCGCTGCATTGCTCCAGACCTGATCGGTATGGGCAAATCCGACAAACCAGACC<br>TGGGTTATTTCTTCGACGACCACGTCCGCTTCATGGATGCCTTCATCGAAGCCCTGGGTCTGGAAGAGG<br>TCGTCCTGGTCATTCACGACTGGGGCTCCGCTCTGGGTTTCCACTGGGCCAAGCGCAATCCAGAGCGC<br>GTCAAAGGTATTGCATTTATGGAGTTCATCCGCCCTATCCCGACCTGGGACGAATGGCCAGAATTTGCCC<br>GCGAGACCTTCCAGGCCTTCCGCACCACCGACGTCGGCCGCAAGCTGATCATCGATCAGAACGTTTTTA<br>TCGAGGGTACGCTGCCGATGGGTGTCGTCCGCCCGCTGACTGAAGTCGAGATGGACCATTACCGCGAG<br>CCGTTCCTGAATCCTGTTGACCGCGAGCCACTGTGGCGCTTCCCAAACGAGCTGCCAATCGCCGGTGAG<br>CCAGCGAACATCGTCGCGCTGGTCGAAGAATACATGGACTGGCTGCACCAGTCCCCTGTCCCGAAGCT<br>GCTGTTCTGGGGCACCCCAGGCGTTCTGATCCCACCGGCCGAAGCCGCTCGCCTGGCCAAAAGCCTGC<br>CTAACTGCAAGGCTGTGGACATCGGCCCGGGTCTGAATCTGCTGCAAGAAGACAACCCGGACCTGATCG<br>GCAGCGAGATCGCGCGCTGGCTGTCTACTCTGGAGATTTCCGGCTCCGAATTCGATTACAAGGACCACG<br>ATGGCGACTATAAGGATCACGACATCGACTACAAGGACGATGACGACAAGTGA
**LAT C26A HaloTag**	ATGGAGGAGGCCATCCTGGTCCCCTGCGTGCTGGGGCTCCTGCTGCTGCCCATCCTGGCCATGTTGAT<br>GGCACTGgccGTGCACTGCCACAGACTGCCAGGATCCGGCGGCTCCGAAATCGGTACTGGCTTTCCATT<br>CGACCCCCATTATGTGGAAGTCCTGGGCGAGCGCATGCACTACGTCGATGTTGGTCCGCGCGATGGCA<br>CCCCTGTGCTGTTCCTGCACGGTAACCCGACCTCCTCCTACGTGTGGCGCAACATCATCCCGCATGTTG<br>CACCGACCCATCGCTGCATTGCTCCAGACCTGATCGGTATGGGCAAATCCGACAAACCAGACCTGGGTT<br>ATTTCTTCGACGACCACGTCCGCTTCATGGATGCCTTCATCGAAGCCCTGGGTCTGGAAGAGGTCGTCC<br>TGGTCATTCACGACTGGGGCTCCGCTCTGGGTTTCCACTGGGCCAAGCGCAATCCAGAGCGCGTCAAA<br>GGTATTGCATTTATGGAGTTCATCCGCCCTATCCCGACCTGGGACGAATGGCCAGAATTTGCCCGCGAG<br>ACCTTCCAGGCCTTCCGCACCACCGACGTCGGCCGCAAGCTGATCATCGATCAGAACGTTTTTATCGAG<br>GGTACGCTGCCGATGGGTGTCGTCCGCCCGCTGACTGAAGTCGAGATGGACCATTACCGCGAGCCGTT<br>CCTGAATCCTGTTGACCGCGAGCCACTGTGGCGCTTCCCAAACGAGCTGCCAATCGCCGGTGAGCCAG<br>CGAACATCGTCGCGCTGGTCGAAGAATACATGGACTGGCTGCACCAGTCCCCTGTCCCGAAGCTGCTGT<br>TCTGGGGCACCCCAGGCGTTCTGATCCCACCGGCCGAAGCCGCTCGCCTGGCCAAAAGCCTGCCTAAC<br>TGCAAGGCTGTGGACATCGGCCCGGGTCTGAATCTGCTGCAAGAAGACAACCCGGACCTGATCGGCAG<br>CGAGATCGCGCGCTGGCTGTCTACTCTGGAGATTTCCGGCTCCGAATTCGATTACAAGGACCACGATGG<br>CGACTATAAGGATCACGACATCGACTACAAGGACGATGACGACAAGTGA
**LAT dCore HaloTag**	ATGGAGGAGGCCATCCTGGTCCCCTGCGTGCTGGGGCTCGCCATGTTGATGGCACTGTGTGTGCACTG<br>CCACAGACTGCCAGGATCCGGCGGCTCCGAAATCGGTACTGGCTTTCCATTCGACCCCCATTATGTGGA<br>AGTCCTGGGCGAGCGCATGCACTACGTCGATGTTGGTCCGCGCGATGGCACCCCTGTGCTGTTCCTGC<br>ACGGTAACCCGACCTCCTCCTACGTGTGGCGCAACATCATCCCGCATGTTGCACCGACCCATCGCTGCA<br>TTGCTCCAGACCTGATCGGTATGGGCAAATCCGACAAACCAGACCTGGGTTATTTCTTCGACGACCACG<br>TCCGCTTCATGGATGCCTTCATCGAAGCCCTGGGTCTGGAAGAGGTCGTCCTGGTCATTCACGACTGGG<br>GCTCCGCTCTGGGTTTCCACTGGGCCAAGCGCAATCCAGAGCGCGTCAAAGGTATTGCATTTATGGAGT<br>TCATCCGCCCTATCCCGACCTGGGACGAATGGCCAGAATTTGCCCGCGAGACCTTCCAGGCCTTCCGCA<br>CCACCGACGTCGGCCGCAAGCTGATCATCGATCAGAACGTTTTTATCGAGGGTACGCTGCCGATGGGTG<br>TCGTCCGCCCGCTGACTGAAGTCGAGATGGACCATTACCGCGAGCCGTTCCTGAATCCTGTTGACCGCG<br>AGCCACTGTGGCGCTTCCCAAACGAGCTGCCAATCGCCGGTGAGCCAGCGAACATCGTCGCGCTGGTC<br>GAAGAATACATGGACTGGCTGCACCAGTCCCCTGTCCCGAAGCTGCTGTTCTGGGGCACCCCAGGCGT<br>TCTGATCCCACCGGCCGAAGCCGCTCGCCTGGCCAAAAGCCTGCCTAACTGCAAGGCTGTGGACATCG<br>GCCCGGGTCTGAATCTGCTGCAAGAAGACAACCCGGACCTGATCGGCAGCGAGATCGCGCGCTGGCTG<br>TCTACTCTGGAGATTTCCGGCTCCGAATTCGATTACAAGGACCACGATGGCGACTATAAGGATCACGAC<br>ATCGACTACAAGGACGATGACGACAAGTGA
**LAT High ASA HaloTag**	ATGGAAGAGGCCATCCTGGTTCTGCTGCTGCTCCTTCTGCTTCTGCTGCCTCTTCTCTTGCTCCTCCTGC<br>TGCTGTGCGTGCACTGTCACAGATTGCCTGGATCCGGCGGCTCCGAAATCGGTACTGGCTTTCCATTCG<br>ACCCCCATTATGTGGAAGTCCTGGGCGAGCGCATGCACTACGTCGATGTTGGTCCGCGCGATGGCACC<br>CCTGTGCTGTTCCTGCACGGTAACCCGACCTCCTCCTACGTGTGGCGCAACATCATCCCGCATGTTGCA<br>CCGACCCATCGCTGCATTGCTCCAGACCTGATCGGTATGGGCAAATCCGACAAACCAGACCTGGGTTAT<br>TTCTTCGACGACCACGTCCGCTTCATGGATGCCTTCATCGAAGCCCTGGGTCTGGAAGAGGTCGTCCTG<br>GTCATTCACGACTGGGGCTCCGCTCTGGGTTTCCACTGGGCCAAGCGCAATCCAGAGCGCGTCAAAGG<br>TATTGCATTTATGGAGTTCATCCGCCCTATCCCGACCTGGGACGAATGGCCAGAATTTGCCCGCGAGAC<br>CTTCCAGGCCTTCCGCACCACCGACGTCGGCCGCAAGCTGATCATCGATCAGAACGTTTTTATCGAGGG<br>TACGCTGCCGATGGGTGTCGTCCGCCCGCTGACTGAAGTCGAGATGGACCATTACCGCGAGCCGTTCC<br>TGAATCCTGTTGACCGCGAGCCACTGTGGCGCTTCCCAAACGAGCTGCCAATCGCCGGTGAGCCAGCG<br>AACATCGTCGCGCTGGTCGAAGAATACATGGACTGGCTGCACCAGTCCCCTGTCCCGAAGCTGCTGTTC<br>TGGGGCACCCCAGGCGTTCTGATCCCACCGGCCGAAGCCGCTCGCCTGGCCAAAAGCCTGCCTAACTG<br>CAAGGCTGTGGACATCGGCCCGGGTCTGAATCTGCTGCAAGAAGACAACCCGGACCTGATCGGCAGCG

	AGATCGCGCGCTGGCTGTCTACTCTGGAGATTTCCGGCTCCGAATTCGATTACAAGGACCACGATGGCG<br>ACTATAAGGATCACGACATCGACTACAAGGACGATGACGACAAGTGA
**24 Å 4TMD RFP**	ATGGGCAGCACCAGAACCGAGATCATCAGAGAGCTGGAAAGAAGCCTGCGCGAGCAGAGAGTGCTGGC<br>CATTTTTCTGCTGGCCCTGCTGATCGTGCTGCTGTGGCTGCTGCAACAGCTGAAAGAGCTGCTGAGAGA<br>ACTGGAACGGCTGCAGAGAGAGGGCAGCTCTGACGAAGATGTGCGGGAACTGCTGCGCGAGATCAAAG<br>AACTGGTGGAAAACATCGTGTACCTGGTTATCATCATCATGGTGCTGGTGCTCGTGATCATTGCCCTGGC<br>CAGAACACAGAAGTACCTGGTCGAGGAACTGAAGCGGCAGGACGGATCCGGCGGCTCCGAAATCGGTA<br>CTGGCTTTCCATTCGACCCCCATTATGTGGAAGTCCTGGGCGAGCGCATGCACTACGTCGATGTTGGTC<br>CGCGCGATGGCACCCCTGTGCTGTTCCTGCACGGTAACCCGACCTCCTCCTACGTGTGGCGCAACATC<br>ATCCCGCATGTTGCACCGACCCATCGCTGCATTGCTCCAGACCTGATCGGTATGGGCAAATCCGACAAA<br>CCAGACCTGGGTTATTTCTTCGACGACCACGTCCGCTTCATGGATGCCTTCATCGAAGCCCTGGGTCTG<br>GAAGAGGTCGTCCTGGTCATTCACGACTGGGGCTCCGCTCTGGGTTTCCACTGGGCCAAGCGCAATCC<br>AGAGCGCGTCAAAGGTATTGCATTTATGGAGTTCATCCGCCCTATCCCGACCTGGGACGAATGGCCAGA<br>ATTTGCCCGCGAGACCTTCCAGGCCTTCCGCACCACCGACGTCGGCCGCAAGCTGATCATCGATCAGA<br>ACGTTTTTATCGAGGGTACGCTGCCGATGGGTGTCGTCCGCCCGCTGACTGAAGTCGAGATGGACCATT<br>ACCGCGAGCCGTTCCTGAATCCTGTTGACCGCGAGCCACTGTGGCGCTTCCCAAACGAGCTGCCAATC<br>GCCGGTGAGCCAGCGAACATCGTCGCGCTGGTCGAAGAATACATGGACTGGCTGCACCAGTCCCCTGT<br>CCCGAAGCTGCTGTTCTGGGGCACCCCAGGCGTTCTGATCCCACCGGCCGAAGCCGCTCGCCTGGCCA<br>AAAGCCTGCCTAACTGCAAGGCTGTGGACATCGGCCCGGGTCTGAATCTGCTGCAAGAAGACAACCCG<br>GACCTGATCGGCAGCGAGATCGCGCGCTGGCTGTCTACTCTGGAGATTTCCGGCTCCGAATTCGATTAC<br>AAGGACCACGATGGCGACTATAAGGATCACGACATCGACTACAAGGACGATGACGACAAGTGA
**32 Å 4TMD RFP**	ATGGGCAGCACCAGAACCGAGATCATCACCCGGCTGAGCTTCAGCCTGCTGCTGCAACTGGTGCTGGC<br>TATCTTTCTGCTGGCCCTGCTGATCGTGCTGCTGTGGCTGCTTCAGCAGCTGAAAGAGCTGCTGAGAGA<br>GCTGGAACGGCTGCAGAGAGAGGGAAGCTCTGACGAGGATGTGCGGGAACTGCTGCGCGAGATCAAA<br>GAACTGGTGGAAAACATCGTGTACCTGGTTATCATCATCATGGTGCTGGTGCTCGTGATCATTGCCCTGG<br>CCGTGCTGCAGATGTACCTCGTCAGGGAACTGAAGCGGCAGGACGGATCCGGCGGCTCCATGGCCTCC<br>TCCGAGGACGTCATCAAGGAGTTCATGCGCTTCAAGGTGCGCATGGAGGGCTCCGTGAACGGCCACGA<br>GTTCGAGATCGAGGGCGAGGGCGAGGGCCGCCCCTACGAGGGCACCCAGACCGCCAAGCTGAAGGTG<br>ACCAAGGGCGGCCCCCTGCCCTTCGCCTGGGACATCCTGTCCCCTCAGTTCCAGTACGGCTCCAAGGC<br>CTACGTGAAGCACCCCGCCGACATCCCCGACTACTTGAAGCTGTCCTTCCCCGAGGGCTTCAAGTGGGA<br>GCGCGTGATGAACTTCGAGGACGGCGGCGTGGTGACCGTGACCCAGGACTCCTCCCTGCAGGACGGC<br>GAGTTCATCTACAAGGTGAAGCTGCGCGGCACCAACTTCCCCTCCGACGGCCCCGTAATGCAGAAGAA<br>GACCATGGGCTGGGAGGCCTCCACCGAGCGGATGTACCCCGAGGACGGCGCCCTGAAGGGCGAGATC<br>AAGATGAGGCTGAAGCTGAAGGACGGCGGCCACTACGACGCCGAGGTCAAGACCACCTACATGGCCAA<br>GAAGCCCGTGCAGCTGCCCGGCGCCTACAAGACCGACATCAAGCTGGACATCACCTCCCACAACGAGG<br>ACTACACCATCGTGGAACAGTACGAGCGCGCCGAGGGCCGCCACTCCACCGGCGCCTCCGGCTCCGA<br>ATTCGATTACAAGGACCACGATGGCGACTATAAGGATCACGACATCGACTACAAGGACGATGACGACAA<br>GTGA
**40 Å 4TMD RFP**	ATGGGCAGCACCAGAACCGAGATCATCACCCGGCTGAGCTTCAGCCTGCTGCTGCAACTGGTGCTGGC<br>TATCTTTCTGCTGGCCCTGCTGATCGTGCTGCTGGTGCTCCTGATCTACCTGAAAGAGCTGCTGAGAGA<br>GCTGGAACGGCTGCAGAGAGAGGGAAGCTCTGACGAGGATGTGCGAGAACTGCTGCGCGAGATCAAGT<br>GGCTGGTCATCGTGATCGTGGCCCTGGTTATCATCATCATGGTGCTGGTCCTGGTCATCATTGCCCTGG<br>CCGTGCTGCAGATGTACCTCGTGCGGGAACTGAAGAGACAGGACGGATCCGGCGGCTCCATGGCCTCC<br>TCCGAGGACGTCATCAAGGAGTTCATGCGCTTCAAGGTGCGCATGGAGGGCTCCGTGAACGGCCACGA<br>GTTCGAGATCGAGGGCGAGGGCGAGGGCCGCCCCTACGAGGGCACCCAGACCGCCAAGCTGAAGGTG<br>ACCAAGGGCGGCCCCCTGCCCTTCGCCTGGGACATCCTGTCCCCTCAGTTCCAGTACGGCTCCAAGGC<br>CTACGTGAAGCACCCCGCCGACATCCCCGACTACTTGAAGCTGTCCTTCCCCGAGGGCTTCAAGTGGGA<br>GCGCGTGATGAACTTCGAGGACGGCGGCGTGGTGACCGTGACCCAGGACTCCTCCCTGCAGGACGGC<br>GAGTTCATCTACAAGGTGAAGCTGCGCGGCACCAACTTCCCCTCCGACGGCCCCGTAATGCAGAAGAA<br>GACCATGGGCTGGGAGGCCTCCACCGAGCGGATGTACCCCGAGGACGGCGCCCTGAAGGGCGAGATC<br>AAGATGAGGCTGAAGCTGAAGGACGGCGGCCACTACGACGCCGAGGTCAAGACCACCTACATGGCCAA<br>GAAGCCCGTGCAGCTGCCCGGCGCCTACAAGACCGACATCAAGCTGGACATCACCTCCCACAACGAGG<br>ACTACACCATCGTGGAACAGTACGAGCGCGCCGAGGGCCGCCACTCCACCGGCGCCTCCGGCTCCGA<br>ATTCGATTACAAGGACCACGATGGCGACTATAAGGATCACGACATCGACTACAAGGACGATGACGACAA<br>GTGA
**24 Å 12TMD RFP**	ATGACCGAGAACGAGATCCGGAAGCTGAGAAAGCTGCTGCGGATCGCTATGTTCCTGCTGGTGTTTCTG<br>CTGATCTGGACCTGGATCAGCCTGGAAACCAGCAAGACCGACGACGACCCTAGCGCTCAGTCTGAAGC<br>TCTGGTGGCCATGAGCCTGATGCTGATTGCCGCCAGCCTGCTGATCATTGCCAAGAGCAAGCTGATGAA<br>GTCCCGGAACGGAGGATCCGGCGGCTCCGAAATCGGTACTGGCTTTCCATTCGACCCCCATTATGTGGA<br>AGTCCTGGGCGAGCGCATGCACTACGTCGATGTTGGTCCGCGCGATGGCACCCCTGTGCTGTTCCTGC<br>ACGGTAACCCGACCTCCTCCTACGTGTGGCGCAACATCATCCCGCATGTTGCACCGACCCATCGCTGCA<br>TTGCTCCAGACCTGATCGGTATGGGCAAATCCGACAAACCAGACCTGGGTTATTTCTTCGACGACCACG<br>TCCGCTTCATGGATGCCTTCATCGAAGCCCTGGGTCTGGAAGAGGTCGTCCTGGTCATTCACGACTGGG<br>GCTCCGCTCTGGGTTTCCACTGGGCCAAGCGCAATCCAGAGCGCGTCAAAGGTATTGCATTTATGGAGT<br>TCATCCGCCCTATCCCGACCTGGGACGAATGGCCAGAATTTGCCCGCGAGACCTTCCAGGCCTTCCGCA<br>CCACCGACGTCGGCCGCAAGCTGATCATCGATCAGAACGTTTTTATCGAGGGTACGCTGCCGATGGGTG<br>TCGTCCGCCCGCTGACTGAAGTCGAGATGGACCATTACCGCGAGCCGTTCCTGAATCCTGTTGACCGCG<br>AGCCACTGTGGCGCTTCCCAAACGAGCTGCCAATCGCCGGTGAGCCAGCGAACATCGTCGCGCTGGTC<br>GAAGAATACATGGACTGGCTGCACCAGTCCCCTGTCCCGAAGCTGCTGTTCTGGGGCACCCCAGGCGT<br>TCTGATCCCACCGGCCGAAGCCGCTCGCCTGGCCAAAAGCCTGCCTAACTGCAAGGCTGTGGACATCG

	GCCCGGGTCTGAATCTGCTGCAAGAAGACAACCCGGACCTGATCGGCAGCGAGATCGCGCGCTGGCTG<br>TCTACTCTGGAGATTTCCGGCTCCGAATTCGATTACAAGGACCACGATGGCGACTATAAGGATCACGAC<br>ATCGACTACAAGGACGATGACGACAAGTGA
**40 Å 12TMD RFP**	ATGACCAAGAAAATCATCATGGTGCTGATCCTGCTGCTGATCATTGCTATGCTGCTGCTGGTGTTCCTGC<br>TGATCGCCACCGTGGTTTCCCTGTGGTGGTCCTGGACCGACGATGATCCTAGCGCCATTTCTGAAGCCC<br>TGGTGGCCATGAGCCTGATGCTGATTGCTGCCAGCCTGCTGATTATCGCCATCAGCAAGCTGCTGAAGT<br>CCAAGAACGGCGGATCCGGCGGCTCCATGGCCTCCTCCGAGGACGTCATCAAGGAGTTCATGCGCTTC<br>AAGGTGCGCATGGAGGGCTCCGTGAACGGCCACGAGTTCGAGATCGAGGGCGAGGGCGAGGGCCGC<br>CCCTACGAGGGCACCCAGACCGCCAAGCTGAAGGTGACCAAGGGCGGCCCCCTGCCCTTCGCCTGGG<br>ACATCCTGTCCCCTCAGTTCCAGTACGGCTCCAAGGCCTACGTGAAGCACCCCGCCGACATCCCCGACT<br>ACTTGAAGCTGTCCTTCCCCGAGGGCTTCAAGTGGGAGCGCGTGATGAACTTCGAGGACGGCGGCGTG<br>GTGACCGTGACCCAGGACTCCTCCCTGCAGGACGGCGAGTTCATCTACAAGGTGAAGCTGCGCGGCAC<br>CAACTTCCCCTCCGACGGCCCCGTAATGCAGAAGAAGACCATGGGCTGGGAGGCCTCCACCGAGCGGA<br>TGTACCCCGAGGACGGCGCCCTGAAGGGCGAGATCAAGATGAGGCTGAAGCTGAAGGACGGCGGCCA<br>CTACGACGCCGAGGTCAAGACCACCTACATGGCCAAGAAGCCCGTGCAGCTGCCCGGCGCCTACAAGA<br>CCGACATCAAGCTGGACATCACCTCCCACAACGAGGACTACACCATCGTGGAACAGTACGAGCGCGCC<br>GAGGGCCGCCACTCCACCGGCGCCTCCGGCTCCGAATTCGATTACAAGGACCACGATGGCGACTATAA<br>GGATCACGACATCGACTACAAGGACGATGACGACAAGTGA
**Soluble HaloTag**	ATGGAATTCGGCGGCTCCGAAATCGGTACTGGCTTTCCATTCGACCCCCATTATGTGGAAGTCCTGGGC<br>GAGCGCATGCACTACGTCGATGTTGGTCCGCGCGATGGCACCCCTGTGCTGTTCCTGCACGGTAACCC<br>GACCTCCTCCTACGTGTGGCGCAACATCATCCCGCATGTTGCACCGACCCATCGCTGCATTGCTCCAGA<br>CCTGATCGGTATGGGCAAATCCGACAAACCAGACCTGGGTTATTTCTTCGACGACCACGTCCGCTTCAT<br>GGATGCCTTCATCGAAGCCCTGGGTCTGGAAGAGGTCGTCCTGGTCATTCACGACTGGGGCTCCGCTC<br>TGGGTTTCCACTGGGCCAAGCGCAATCCAGAGCGCGTCAAAGGTATTGCATTTATGGAGTTCATCCGCC<br>CTATCCCGACCTGGGACGAATGGCCAGAATTTGCCCGCGAGACCTTCCAGGCCTTCCGCACCACCGAC<br>GTCGGCCGCAAGCTGATCATCGATCAGAACGTTTTTATCGAGGGTACGCTGCCGATGGGTGTCGTCCGC<br>CCGCTGACTGAAGTCGAGATGGACCATTACCGCGAGCCGTTCCTGAATCCTGTTGACCGCGAGCCACTG<br>TGGCGCTTCCCAAACGAGCTGCCAATCGCCGGTGAGCCAGCGAACATCGTCGCGCTGGTCGAAGAATA<br>CATGGACTGGCTGCACCAGTCCCCTGTCCCGAAGCTGCTGTTCTGGGGCACCCCAGGCGTTCTGATCC<br>CACCGGCCGAAGCCGCTCGCCTGGCCAAAAGCCTGCCTAACTGCAAGGCTGTGGACATCGGCCCGGG<br>TCTGAATCTGCTGCAAGAAGACAACCCGGACCTGATCGGCAGCGAGATCGCGCGCTGGCTGTCTACTCT<br>GGAGATTTCCGGCTCCGGATCCGACTACAAGGACGATGACGACAAGTGA
**M HaloTag**	ATGGGCAGTTCTAAGAGCAAGCCCAAGGATCCCAGCCAGCGGCGGAACAACAACAATGGACCTGTGGC<br>CACTGAATTCGGCGGCTCCGAAATCGGTACTGGCTTTCCATTCGACCCCCATTATGTGGAAGTCCTGGG<br>CGAGCGCATGCACTACGTCGATGTTGGTCCGCGCGATGGCACCCCTGTGCTGTTCCTGCACGGTAACC<br>CGACCTCCTCCTACGTGTGGCGCAACATCATCCCGCATGTTGCACCGACCCATCGCTGCATTGCTCCAG<br>ACCTGATCGGTATGGGCAAATCCGACAAACCAGACCTGGGTTATTTCTTCGACGACCACGTCCGCTTCAT<br>GGATGCCTTCATCGAAGCCCTGGGTCTGGAAGAGGTCGTCCTGGTCATTCACGACTGGGGCTCCGCTC<br>TGGGTTTCCACTGGGCCAAGCGCAATCCAGAGCGCGTCAAAGGTATTGCATTTATGGAGTTCATCCGCC<br>CTATCCCGACCTGGGACGAATGGCCAGAATTTGCCCGCGAGACCTTCCAGGCCTTCCGCACCACCGAC<br>GTCGGCCGCAAGCTGATCATCGATCAGAACGTTTTTATCGAGGGTACGCTGCCGATGGGTGTCGTCCGC<br>CCGCTGACTGAAGTCGAGATGGACCATTACCGCGAGCCGTTCCTGAATCCTGTTGACCGCGAGCCACTG<br>TGGCGCTTCCCAAACGAGCTGCCAATCGCCGGTGAGCCAGCGAACATCGTCGCGCTGGTCGAAGAATA<br>CATGGACTGGCTGCACCAGTCCCCTGTCCCGAAGCTGCTGTTCTGGGGCACCCCAGGCGTTCTGATCC<br>CACCGGCCGAAGCCGCTCGCCTGGCCAAAAGCCTGCCTAACTGCAAGGCTGTGGACATCGGCCCGGG<br>TCTGAATCTGCTGCAAGAAGACAACCCGGACCTGATCGGCAGCGAGATCGCGCGCTGGCTGTCTACTCT<br>GGAGATTTCCGGCTCCGGATCCGACTACAAGGACGATGACGACAAGTGA
**PM HaloTag**	ATGGGCTGCATCAAGAGCAAGCGGAAGGACAAGGACCTGGAACTGAAGCTGCGGATCCTGCAGAGCAC<br>CGTGCCTAGAGCTAGAGATCCTCCAGTGGCCACAGAATTCGGCGGCTCCGAAATCGGTACTGGCTTTCC<br>ATTCGACCCCCATTATGTGGAAGTCCTGGGCGAGCGCATGCACTACGTCGATGTTGGTCCGCGCGATG<br>GCACCCCTGTGCTGTTCCTGCACGGTAACCCGACCTCCTCCTACGTGTGGCGCAACATCATCCCGCATG<br>TTGCACCGACCCATCGCTGCATTGCTCCAGACCTGATCGGTATGGGCAAATCCGACAAACCAGACCTGG<br>GTTATTTCTTCGACGACCACGTCCGCTTCATGGATGCCTTCATCGAAGCCCTGGGTCTGGAAGAGGTCG<br>TCCTGGTCATTCACGACTGGGGCTCCGCTCTGGGTTTCCACTGGGCCAAGCGCAATCCAGAGCGCGTC<br>AAAGGTATTGCATTTATGGAGTTCATCCGCCCTATCCCGACCTGGGACGAATGGCCAGAATTTGCCCGC<br>GAGACCTTCCAGGCCTTCCGCACCACCGACGTCGGCCGCAAGCTGATCATCGATCAGAACGTTTTTATC<br>GAGGGTACGCTGCCGATGGGTGTCGTCCGCCCGCTGACTGAAGTCGAGATGGACCATTACCGCGAGCC<br>GTTCCTGAATCCTGTTGACCGCGAGCCACTGTGGCGCTTCCCAAACGAGCTGCCAATCGCCGGTGAGC<br>CAGCGAACATCGTCGCGCTGGTCGAAGAATACATGGACTGGCTGCACCAGTCCCCTGTCCCGAAGCTG<br>CTGTTCTGGGGCACCCCAGGCGTTCTGATCCCACCGGCCGAAGCCGCTCGCCTGGCCAAAAGCCTGCC<br>TAACTGCAAGGCTGTGGACATCGGCCCGGGTCTGAATCTGCTGCAAGAAGACAACCCGGACCTGATCG<br>GCAGCGAGATCGCGCGCTGGCTGTCTACTCTGGAGATTTCCGGCTCCGGATCCGACTACAAGGACGAT<br>GACGACAAGTGA
**G HaloTag**	ATGGACTACAAGGACGATGACGACAAGGAATTCGGCGGCTCCGAAATCGGTACTGGCTTTCCATTCGAC<br>CCCCATTATGTGGAAGTCCTGGGCGAGCGCATGCACTACGTCGATGTTGGTCCGCGCGATGGCACCCC<br>TGTGCTGTTCCTGCACGGTAACCCGACCTCCTCCTACGTGTGGCGCAACATCATCCCGCATGTTGCACC<br>GACCCATCGCTGCATTGCTCCAGACCTGATCGGTATGGGCAAATCCGACAAACCAGACCTGGGTTATTT<br>CTTCGACGACCACGTCCGCTTCATGGATGCCTTCATCGAAGCCCTGGGTCTGGAAGAGGTCGTCCTGGT<br>CATTCACGACTGGGGCTCCGCTCTGGGTTTCCACTGGGCCAAGCGCAATCCAGAGCGCGTCAAAGGTA<br>TTGCATTTATGGAGTTCATCCGCCCTATCCCGACCTGGGACGAATGGCCAGAATTTGCCCGCGAGACCT

	TCCAGGCCTTCCGCACCACCGACGTCGGCCGCAAGCTGATCATCGATCAGAACGTTTTTATCGAGGGTA<br>CGCTGCCGATGGGTGTCGTCCGCCCGCTGACTGAAGTCGAGATGGACCATTACCGCGAGCCGTTCCTG<br>AATCCTGTTGACCGCGAGCCACTGTGGCGCTTCCCAAACGAGCTGCCAATCGCCGGTGAGCCAGCGAA<br>CATCGTCGCGCTGGTCGAAGAATACATGGACTGGCTGCACCAGTCCCCTGTCCCGAAGCTGCTGTTCTG<br>GGGCACCCCAGGCGTTCTGATCCCACCGGCCGAAGCCGCTCGCCTGGCCAAAAGCCTGCCTAACTGCA<br>AGGCTGTGGACATCGGCCCGGGTCTGAATCTGCTGCAAGAAGACAACCCGGACCTGATCGGCAGCGAG<br>ATCGCGCGCTGGCTGTCTACTCTGGAGATTTCCGGCTCCGGATCCGGTGGTAGCTTTCGTAGTGATGGC<br>AAAAAGAAAAAGAAGAAATCCAAGACCAAATGCCAGCTGCTGTGA
**PPF HaloTag**	ATGGACTACAAGGACGATGACGACAAGGAATTCGGCGGCTCCGAAATCGGTACTGGCTTTCCATTCGAC<br>CCCCATTATGTGGAAGTCCTGGGCGAGCGCATGCACTACGTCGATGTTGGTCCGCGCGATGGCACCCC<br>TGTGCTGTTCCTGCACGGTAACCCGACCTCCTCCTACGTGTGGCGCAACATCATCCCGCATGTTGCACC<br>GACCCATCGCTGCATTGCTCCAGACCTGATCGGTATGGGCAAATCCGACAAACCAGACCTGGGTTATTT<br>CTTCGACGACCACGTCCGCTTCATGGATGCCTTCATCGAAGCCCTGGGTCTGGAAGAGGTCGTCCTGGT<br>CATTCACGACTGGGGCTCCGCTCTGGGTTTCCACTGGGCCAAGCGCAATCCAGAGCGCGTCAAAGGTA<br>TTGCATTTATGGAGTTCATCCGCCCTATCCCGACCTGGGACGAATGGCCAGAATTTGCCCGCGAGACCT<br>TCCAGGCCTTCCGCACCACCGACGTCGGCCGCAAGCTGATCATCGATCAGAACGTTTTTATCGAGGGTA<br>CGCTGCCGATGGGTGTCGTCCGCCCGCTGACTGAAGTCGAGATGGACCATTACCGCGAGCCGTTCCTG<br>AATCCTGTTGACCGCGAGCCACTGTGGCGCTTCCCAAACGAGCTGCCAATCGCCGGTGAGCCAGCGAA<br>CATCGTCGCGCTGGTCGAAGAATACATGGACTGGCTGCACCAGTCCCCTGTCCCGAAGCTGCTGTTCTG<br>GGGCACCCCAGGCGTTCTGATCCCACCGGCCGAAGCCGCTCGCCTGGCCAAAAGCCTGCCTAACTGCA<br>AGGCTGTGGACATCGGCCCGGGTCTGAATCTGCTGCAAGAAGACAACCCGGACCTGATCGGCAGCGAG<br>ATCGCGCGCTGGCTGTCTACTCTGGAGATTTCCGGCTCCGGATCCGGTGGTAGCATGAAAAAGCATCGT<br>TGTAAATGTTGCAGCATTATGTGA

*The WT-LAT HaloTag construct contains a 5mer glycine-serine linker between the C-terminal of the LAT region used here and the N-terminal of HaloTag. All other HaloTag constructs contain a 3mer glycine-serine linker at this location.

Supplementary Table 6. DNA sequences for transcription factor delivery studies.

Construct	DNA sequence
**Soluble synTF**	TGGAATTCGGCGGCTCCCCTAAGAAAAAGCGCAAAGTCTCCGGAAGCCAGTACCTGCCTGACACCGA<br>CGACCGGCACAGAATCGAGGAAAAGCGGAAGCGGACCTACGAGACATTCAAGAGCATCATGAAGAAG<br>TCCCCATTCAGCGGCCCCACCGATCCTAGACCTCCACCTAGAAGAATCGCCGTGCCTAGCAGATCCA<br>GCGCCTCTGTGCCTAAACCTGCTCCTCAGCCTTATCCTTTCACCAGCAGCCTGAGCACCATCAACTAC<br>GACGAGTTCCCCACAATGGTGTTCCCCAGCGGACAGATCAGCCAGGCTTCTGCTCTTGCTCCAGCTC<br>CTCCTCAGGTTCTGCCTCAAGCTCCTGCTCCGGCTCCAGCACCAGCTATGGTTTCTGCTTTGGCCCAG<br>GCTCCTGCACCTGTGCCTGTTCTTGCTCCTGGACCACCTCAGGCTGTTGCTCCACCAGCTCCTAAACC<br>TACACAGGCCGGCGAGGGAACACTGTCTGAAGCCCTGCTGCAACTCCAGTTCGACGACGAGGATCTG<br>GGAGCACTGCTGGGCAATAGCACAGACCCTGCCGTGTTTACCGATCTGGCCAGCGTGGACAACAGCG<br>AGTTTCAGCAGCTCCTGAACCAGGGCATCCCTGTGGCTCCTCACACCACAGAGCCCATGCTGATGGA<br>ATACCCCGAGGCCATCACCAGACTGGTCACCGGCGCTCAAAGACCTCCAGATCCTGCACCAGCACCT<br>CTTGGAGCACCTGGCCTGCCTAATGGACTGCTGAGCGGAGATGAGGACTTCAGCTCTATCGCCGACA<br>TGGATTTCAGCGCCCTGCTCGgttccGGTGGTGGCGGGTCTGGAGGAGGAGGTAGTGGTGGAGGTGGC<br>TCTggtacCGCTAGACCCGGCGAAAGACCTTTCCAGTGCCGGATCTGCATGAGGAACTTCAGCAAGGGC<br>GAGAGACTCGTGCGGCACACCAGAACACACACAGGCGAGAAGCCCTTCCAGTGTAGAATCTGTATGC<br>GCAACTTCAGCCGGATGGACAACCTGAGCACCCACCTGAGAACCCATACCGGGGAGAAGCCATTTCA<br>ATGCCGCATCTGTATGAGAAATTTTTCCCGGAAGGACGCCCTGAACCGGCACCTGAAAACACACCTGA<br>GAGGCAGCGGCTCCGGATCCGACTACAAGGACGATGACGACAAGTGA
**M synTF**	ATGGGCAGTTCTAAGAGCAAGCCCAAGGAcCCtAGCCAGCGGCGGAACAACAACAATGGACCTGTGG<br>CCACTGAATTCGGCGGCTCCCCTAAGAAAAAGCGCAAAGTCTCCGGAAGCCAGTACCTGCCTGACAC<br>CGACGACCGGCACAGAATCGAGGAAAAGCGGAAGCGGACCTACGAGACATTCAAGAGCATCATGAAG<br>AAGTCCCCATTCAGCGGCCCCACCGATCCTAGACCTCCACCTAGAAGAATCGCCGTGCCTAGCAGAT<br>CCAGCGCCTCTGTGCCTAAACCTGCTCCTCAGCCTTATCCTTTCACCAGCAGCCTGAGCACCATCAAC<br>TACGACGAGTTCCCCACAATGGTGTTCCCCAGCGGACAGATCAGCCAGGCTTCTGCTCTTGCTCCAG<br>CTCCTCCTCAGGTTCTGCCTCAAGCTCCTGCTCCGGCTCCAGCACCAGCTATGGTTTCTGCTTTGGCC<br>CAGGCTCCTGCACCTGTGCCTGTTCTTGCTCCTGGACCACCTCAGGCTGTTGCTCCACCAGCTCCTAA<br>ACCTACACAGGCCGGCGAGGGAACACTGTCTGAAGCCCTGCTGCAACTCCAGTTCGACGACGAGGAT<br>CTGGGAGCACTGCTGGGCAATAGCACAGACCCTGCCGTGTTTACCGATCTGGCCAGCGTGGACAACA<br>GCGAGTTTCAGCAGCTCCTGAACCAGGGCATCCCTGTGGCTCCTCACACCACAGAGCCCATGCTGAT<br>GGAATACCCCGAGGCCATCACCAGACTGGTCACCGGCGCTCAAAGACCTCCAGATCCTGCACCAGCA<br>CCTCTTGGAGCACCTGGCCTGCCTAATGGACTGCTGAGCGGAGATGAGGACTTCAGCTCTATCGCCG<br>ACATGGATTTCAGCGCCCTGCTCGgttccGGTGGTGGCGGGTCTGGAGGAGGAGGTAGTGGTGGAGGT<br>GGCTCTggtacCGCTAGACCCGGCGAAAGACCTTTCCAGTGCCGGATCTGCATGAGGAACTTCAGCAAG<br>GGCGAGAGACTCGTGCGGCACACCAGAACACACACAGGCGAGAAGCCCTTCCAGTGTAGAATCTGTA<br>TGCGCAACTTCAGCCGGATGGACAACCTGAGCACCCACCTGAGAACCCATACCGGGGAGAAGCCATT<br>TCAATGCCGCATCTGTATGAGAAATTTTTCCCGGAAGGACGCCCTGAACCGGCACCTGAAAACACACC<br>TGAGAGGCAGCGGCTCCGGATCCGACTACAAGGACGATGACGACAAGTGA
**PM synTF**	ATGGGCTGCATCAAGAGCAAGCGGAAGGACAAGGACCTGGAACTGAAGCTGagaATCCTGCAGAGCA<br>CCGTGCCTAGAGCTAGAGATCCTCCAGTGGCCACAGAATTCGGCGGCTCCCCTAAGAAAAAGCGCAA<br>AGTCTCCGGAAGCCAGTACCTGCCTGACACCGACGACCGGCACAGAATCGAGGAAAAGCGGAAGCG<br>GACCTACGAGACATTCAAGAGCATCATGAAGAAGTCCCCATTCAGCGGCCCCACCGATCCTAGACCTC<br>CACCTAGAAGAATCGCCGTGCCTAGCAGATCCAGCGCCTCTGTGCCTAAACCTGCTCCTCAGCCTTAT<br>CCTTTCACCAGCAGCCTGAGCACCATCAACTACGACGAGTTCCCCACAATGGTGTTCCCCAGCGGAC<br>AGATCAGCCAGGCTTCTGCTCTTGCTCCAGCTCCTCCTCAGGTTCTGCCTCAAGCTCCTGCTCCGGCT<br>CCAGCACCAGCTATGGTTTCTGCTTTGGCCCAGGCTCCTGCACCTGTGCCTGTTCTTGCTCCTGGACC<br>ACCTCAGGCTGTTGCTCCACCAGCTCCTAAACCTACACAGGCCGGCGAGGGAACACTGTCTGAAGCC<br>CTGCTGCAACTCCAGTTCGACGACGAGGATCTGGGAGCACTGCTGGGCAATAGCACAGACCCTGCCG<br>TGTTTACCGATCTGGCCAGCGTGGACAACAGCGAGTTTCAGCAGCTCCTGAACCAGGGCATCCCTGT<br>GGCTCCTCACACCACAGAGCCCATGCTGATGGAATACCCCGAGGCCATCACCAGACTGGTCACCGGC<br>GCTCAAAGACCTCCAGATCCTGCACCAGCACCTCTTGGAGCACCTGGCCTGCCTAATGGACTGCTGA<br>GCGGAGATGAGGACTTCAGCTCTATCGCCGACATGGATTTCAGCGCCCTGCTCGgttccGGTGGTGGCG<br>GGTCTGGAGGAGGAGGTAGTGGTGGAGGTGGCTCTggtacCGCTAGACCCGGCGAAAGACCTTTCCAG<br>TGCCGGATCTGCATGAGGAACTTCAGCAAGGGCGAGAGACTCGTGCGGCACACCAGAACACACACAG<br>GCGAGAAGCCCTTCCAGTGTAGAATCTGTATGCGCAACTTCAGCCGGATGGACAACCTGAGCACCCA<br>CCTGAGAACCCATACCGGGGAGAAGCCATTTCAATGCCGCATCTGTATGAGAAATTTTTCCCGGAAGG<br>ACGCCCTGAACCGGCACCTGAAAACACACCTGAGAGGCAGCGGCTCCGGATCCGACTACAAGGACG<br>ATGACGACAAGTGA
**G synTF**	ATGGACTACAAGGACGATGACGACAAGGAATTCGGCGGCTCCCCTAAGAAAAAGCGCAAAGTCTCCG<br>GAAGCCAGTACCTGCCTGACACCGACGACCGGCACAGAATCGAGGAAAAGCGGAAGCGGACCTACG<br>AGACATTCAAGAGCATCATGAAGAAGTCCCCATTCAGCGGCCCCACCGATCCTAGACCTCCACCTAGA<br>AGAATCGCCGTGCCTAGCAGATCCAGCGCCTCTGTGCCTAAACCTGCTCCTCAGCCTTATCCTTTCAC<br>CAGCAGCCTGAGCACCATCAACTACGACGAGTTCCCCACAATGGTGTTCCCCAGCGGACAGATCAGC<br>CAGGCTTCTGCTCTTGCTCCAGCTCCTCCTCAGGTTCTGCCTCAAGCTCCTGCTCCGGCTCCAGCACC<br>AGCTATGGTTTCTGCTTTGGCCCAGGCTCCTGCACCTGTGCCTGTTCTTGCTCCTGGACCACCTCAGG

	CTGTTGCTCCACCAGCTCCTAAACCTACACAGGCCGGCGAGGGAACACTGTCTGAAGCCCTGCTGCA<br>ACTCCAGTTCGACGACGAGGATCTGGGAGCACTGCTGGGCAATAGCACAGACCCTGCCGTGTTTACC<br>GATCTGGCCAGCGTGGACAACAGCGAGTTTCAGCAGCTCCTGAACCAGGGCATCCCTGTGGCTCCTC<br>ACACCACAGAGCCCATGCTGATGGAATACCCCGAGGCCATCACCAGACTGGTCACCGGCGCTCAAAG<br>ACCTCCAGATCCTGCACCAGCACCTCTTGGAGCACCTGGCCTGCCTAATGGACTGCTGAGCGGAGAT<br>GAGGACTTCAGCTCTATCGCCGACATGGATTTCAGCGCCCTGCTCGgttccGGTGGTGGCGGGTCTGGA<br>GGAGGAGGTAGTGGTGGAGGTGGCTCTggtacCGCTAGACCCGGCGAAAGACCTTTCCAGTGCCGGAT<br>CTGCATGAGGAACTTCAGCAAGGGCGAGAGACTCGTGCGGCACACCAGAACACACACAGGCGAGAA<br>GCCCTTCCAGTGTAGAATCTGTATGCGCAACTTCAGCCGGATGGACAACCTGAGCACCCACCTGAGA<br>ACCCATACCGGGGAGAAGCCATTTCAATGCCGCATCTGTATGAGAAATTTTTCCCGGAAGGACGCCCT<br>GAACCGGCACCTGAAAACACACCTGAGAGGCAGCGGCTCCGGATCCGGTGGTAGCTTTCGTAGTGAT<br>GGCAAAAAGAAAAAGAAGAAATCCAAGACCAAATGCCAGCTGCTGTGA
**PPF synTF**	ATGGACTACAAGGACGATGACGACAAGGAATTCGGCGGCTCCCCTAAGAAAAAGCGCAAAGTCTCCG<br>GAAGCCAGTACCTGCCTGACACCGACGACCGGCACAGAATCGAGGAAAAGCGGAAGCGGACCTACG<br>AGACATTCAAGAGCATCATGAAGAAGTCCCCATTCAGCGGCCCCACCGATCCTAGACCTCCACCTAGA<br>AGAATCGCCGTGCCTAGCAGATCCAGCGCCTCTGTGCCTAAACCTGCTCCTCAGCCTTATCCTTTCAC<br>CAGCAGCCTGAGCACCATCAACTACGACGAGTTCCCCACAATGGTGTTCCCCAGCGGACAGATCAGC<br>CAGGCTTCTGCTCTTGCTCCAGCTCCTCCTCAGGTTCTGCCTCAAGCTCCTGCTCCGGCTCCAGCACC<br>AGCTATGGTTTCTGCTTTGGCCCAGGCTCCTGCACCTGTGCCTGTTCTTGCTCCTGGACCACCTCAGG<br>CTGTTGCTCCACCAGCTCCTAAACCTACACAGGCCGGCGAGGGAACACTGTCTGAAGCCCTGCTGCA<br>ACTCCAGTTCGACGACGAGGATCTGGGAGCACTGCTGGGCAATAGCACAGACCCTGCCGTGTTTACC<br>GATCTGGCCAGCGTGGACAACAGCGAGTTTCAGCAGCTCCTGAACCAGGGCATCCCTGTGGCTCCTC<br>ACACCACAGAGCCCATGCTGATGGAATACCCCGAGGCCATCACCAGACTGGTCACCGGCGCTCAAAG<br>ACCTCCAGATCCTGCACCAGCACCTCTTGGAGCACCTGGCCTGCCTAATGGACTGCTGAGCGGAGAT<br>GAGGACTTCAGCTCTATCGCCGACATGGATTTCAGCGCCCTGCTCGgttccGGTGGTGGCGGGTCTGGA<br>GGAGGAGGTAGTGGTGGAGGTGGCTCTggtacCGCTAGACCCGGCGAAAGACCTTTCCAGTGCCGGAT<br>CTGCATGAGGAACTTCAGCAAGGGCGAGAGACTCGTGCGGCACACCAGAACACACACAGGCGAGAA<br>GCCCTTCCAGTGTAGAATCTGTATGCGCAACTTCAGCCGGATGGACAACCTGAGCACCCACCTGAGA<br>ACCCATACCGGGGAGAAGCCATTTCAATGCCGCATCTGTATGAGAAATTTTTCCCGGAAGGACGCCCT<br>GAACCGGCACCTGAAAACACACCTGAGAGGCAGCGGCTCCGGATCCGGTGGTAGCATGAAAAAGCAT<br>CGTTGTAAATGTTGCAGCATTATGTGA

Supplementary Table 7. Western blot antibodies used in this study and associated sample preparation considerations.

Target	Supplier (#)	Denature temperature / time	Antibody dilution	Reducing/Non-reducing Laemmli	Animal of origin
HaloTag TMR Ligand	Promega (G8251)	70°C / 3 min	N/A	Reducing (β-mercaptoethanol [BME])	N/A
FLAG tag	Sigma (F1804)	70°C / 10 min	1:1000	Reducing (BME)	Mouse
CD9	Santa Cruz (sc-13118)	95°C / 10 min	1:500	Reducing (dithiothreitol [DTT])	Mouse
CD81	Santa Cruz (sc-23962)	95°C / 10 min	1:500	Non-reducing	Mouse
Alix	Abcam (Ab117600)	95°C / 10 min	1:500	Reducing (DTT)	Mouse
Calnexin	Abcam (Ab22595)	70°C / 10 min	1:1000	Reducing (DTT)	Rabbit
VSV-G	Abcam (ab50549)	95°C / 5 min	1:1000	Reducing (DTT)	Mouse
Rabbit	Invitrogen (32460)	Not applicable	1:3000	Not applicable	Goat
Mouse	Cell Signaling Technology (7076)	Not applicable	1:3000	Not applicable	Horse

Supplementary Table 8. BD Fortessa flow cytometry lasers and photomultiplier tube (PMT) filter sets for specific fluorophores evaluated in this study.

Fluorophore	Channel name	Excitation laser (nm)	Filter set*
eBFP2	Pacific Blue	405	450/50
dsRed-Express2	PE-Texas Red	552	610/20; 600LP
miRFP720	Alexa 750	685	730/45;690 LP

*LP, Long pass

Supplementary Table 9. Summary of statistical tests used in this study and their assumptions.

Figure	Statistical Test	Assumptions		
		**n are independent?**	**Homogeneity of variances met?**	**Normally distributed residuals met?**
1c	1-way ANOVA + Sidak	Yes	*Yes, Brown-Forsythe*	*Yes, D'Agostino-Pearson*
1d	Unpaired t test	Yes	*Yes, F test*	*Yes, Shapiro-Wilk*
2a	Kruskal-Wallis	Yes	*Not required for test*	*Not required for test*
2b	Kruskal-Wallis	Yes	*Not required for test*	*Not required for test*
2c	Kruskal-Wallis	Yes	*Not required for test*	*Not required for test*
2d	Kruskal-Wallis	Yes	*Not required for test*	*Not required for test*
2e	Kruskal-Wallis	Yes	*Not required for test*	*Not required for test*
2f	Kruskal-Wallis	Yes	*Not required for test*	*Not required for test*
2g	Kruskal-Wallis	Yes	*Not required for test*	*Not required for test*
S5a	Kruskal-Wallis	Yes	*Not required for test*	*Not required for test*
S5b	Kruskal-Wallis	Yes	*Not required for test*	*Not required for test*
S5c	Kruskal-Wallis	Yes	*Not required for test*	*Not required for test*
S5d	Kruskal-Wallis	Yes	*Not required for test*	*Not required for test*
S5e	Kruskal-Wallis	Yes	*Not required for test*	*Not required for test*
S5f	Kruskal-Wallis	Yes	*Not required for test*	*Not required for test*
S5h	Kruskal-Wallis	Yes	*Not required for test*	*Not required for test*
S6a	Kruskal-Wallis	Yes	*Not required for test*	*Not required for test*
S6b	Kruskal-Wallis	Yes	*Not required for test*	*Not required for test*
S6c	Kruskal-Wallis	Yes	*Not required for test*	*Not required for test*
S6d	Kruskal-Wallis	Yes	*Not required for test*	*Not required for test*
S6e	Kruskal-Wallis	Yes	*Not required for test*	*Not required for test*
S6f	Kruskal-Wallis	Yes	*Not required for test*	*Not required for test*
S6g	Kruskal-Wallis	Yes	*Not required for test*	*Not required for test*
S7a	Kruskal-Wallis	Yes	*Not required for test*	*Not required for test*
S7b	Kruskal-Wallis	Yes	*Not required for test*	*Not required for test*
S7c	Kruskal-Wallis	Yes	*Not required for test*	*Not required for test*
S7d	Kruskal-Wallis	Yes	*Not required for test*	*Not required for test*
S7e	Kruskal-Wallis	Yes	*Not required for test*	*Not required for test*
S7f	Kruskal-Wallis	Yes	*Not required for test*	*Not required for test*
S7g	Kruskal-Wallis	Yes	*Not required for test*	*Not required for test*
S8a	Kruskal-Wallis	Yes	*Not required for test*	*Not required for test*
S8b	Kruskal-Wallis	Yes	*Not required for test*	*Not required for test*
S8c	Kruskal-Wallis	Yes	*Not required for test*	*Not required for test*
S8d	Kruskal-Wallis	Yes	*Not required for test*	*Not required for test*
S8e	Kruskal-Wallis	Yes	*Not required for test*	*Not required for test*
S8f	Kruskal-Wallis	Yes	*Not required for test*	*Not required for test*
S8h	Kruskal-Wallis	Yes	*Not required for test*	*Not required for test*
S9a	Kruskal-Wallis	Yes	*Not required for test*	*Not required for test*
S9b	Kruskal-Wallis	Yes	*Not required for test*	*Not required for test*
S9c	Kruskal-Wallis	Yes	*Not required for test*	*Not required for test*
S9d	Kruskal-Wallis	Yes	*Not required for test*	*Not required for test*
S9e	Kruskal-Wallis	Yes	*Not required for test*	*Not required for test*
S9f	Kruskal-Wallis	Yes	*Not required for test*	*Not required for test*
S10a	Kruskal-Wallis	Yes	*Not required for test*	*Not required for test*

S10b	Kruskal-Wallis	Yes	*Not required for test*	*Not required for test*
S10c	Kruskal-Wallis	Yes	*Not required for test*	*Not required for test*
S10d	Kruskal-Wallis	Yes	*Not required for test*	*Not required for test*
S10e	Kruskal-Wallis	Yes	*Not required for test*	*Not required for test*
S10f	Kruskal-Wallis	Yes	*Not required for test*	*Not required for test*
S10g	Kruskal-Wallis	Yes	*Not required for test*	*Not required for test*
3c	1-way ANOVA + Tukey	Yes	*Yes, Brown-Forsythe*	*Yes, D'Agostino-Pearson*
3e	Welch's ANOVA + Dunnett T3	Yes	*Not required for test*	*Yes, D'Agostino-Pearson*
3f	2-way ANOVA + Dunnett	Yes	*Yes, Spearman*	*Yes, D'Agostino-Pearson*
3g	2-way ANOVA + Dunnett	Yes	*Yes, Spearman*	*Yes, D'Agostino-Pearson*
S13a	Ordinary 2-way ANOVA	Yes	*Yes, Spearman*	*Yes, Kolmogorov-Smirnov*
4b,4TMD	1-way ANOVA + Tukey	Yes	*Yes, Brown-Forsythe*	*Yes, D'Agostino-Pearson*
4b,12TMD	Unpaired t test	Yes	*Yes, F test*	*Yes, D'Agostino-Pearson*
4c	1-way ANOVA + Tukey	Yes	*Yes, Brown-Forsythe*	*Yes, Shapiro-Wilk*
4d,4TMD	2-way ANOVA + Tukey	*Yes*	*Yes, Spearman*	*Yes, D'Agostino-Pearson*
4d,12TMD	2-way ANOVA + Tukey	*Yes*	*Yes, Spearman*	*Yes, D'Agostino-Pearson*
4e,4TMD	2-way ANOVA + Tukey	*Yes*	*Yes, Spearman*	*Yes, D'Agostino-Pearson*
4e,12TMD	2-way ANOVA + Tukey	*Yes*	*Yes, Spearman*	*Yes, D'Agostino-Pearson*
S13b	Ordinary 2-way ANOVA	Yes	*Yes, Spearman*	*Yes, Kolmogorov-Smirnov*
5b	Welch's ANOVA + Dunnett T3	Yes	*Not required for test*	*Yes, D'Agostino-Pearson*
5c	1-way ANOVA + Tukey	Yes	*Yes, Brown-Forsythe*	*Yes, D'Agostino-Pearson*
5d	*2-way ANOVA + Dunnett*	*Yes*	*Yes, Spearman*	*Yes, Anderson-Darling*
S13c	Ordinary 2-way ANOVA	Yes	*Yes, Spearman*	*Yes, D'Agostino-Pearson*
7b	*1-way ANOVA of log transform + Dunnett*	*Yes*	*Yes, Brown-Forsythe*	*Yes, D'Agostino-Pearson*
7c	*1-way ANOVA + Dunnett*	*Yes*	*Yes, Brown-Forsythe*	*No per D'Agostino-Pearson, but justifying test use because residuals are expected to be Gaussian (see 5d)*
7f	*1-way ANOVA + Dunnett*	*Yes*	*Yes, Brown-Forsythe*	*Yes, D'Agostino-Pearson*
