# Supplementary material for: Enhancing extracellular vesicle cargo loading and functional delivery by engineering protein-lipid interactions
Source: Nat Commun. 2024 Jul 4;15:5618. doi: 10.1038/s41467-024-49678-z (PMC11224323; doi:10.1038/s41467-024-49678-z)
Supplement: Supplementary file 3 — Reporting Summary [file 41467_2024_49678_MOESM3_ESM.pdf]

nature portfolio

Corresponding author(s): Neha Kamat and Josh Leonard

Last updated by author(s): May 5, 2024

Reporting Summary

Nature Portfolio wishes to improve the reproducibility of the work that we publish. This form provides structure for consistency and transparency in reporting. For further information on Nature Portfolio policies, see our Editorial Policies and the Editorial Policy Checklist.

Statistics

For all statistical analyses, confirm that the following items are present in the figure legend, table legend, main text, or Methods section.

n/a	Confirmed	
☐	☒	The exact sample size (*n*) for each experimental group/condition, given as a discrete number and unit of measurement
☐	☒	A statement on whether measurements were taken from distinct samples or whether the same sample was measured repeatedly
☐	☒	The statistical test(s) used AND whether they are one- or two-sided<br>*Only common tests should be described solely by name; describe more complex techniques in the Methods section.*
☒	☐	A description of all covariates tested
☐	☒	A description of any assumptions or corrections, such as tests of normality and adjustment for multiple comparisons
☐	☒	A full description of the statistical parameters including central tendency (e.g. means) or other basic estimates (e.g. regression coefficient) AND variation (e.g. standard deviation) or associated estimates of uncertainty (e.g. confidence intervals)
☐	☒	For null hypothesis testing, the test statistic (e.g. F, t, r) with confidence intervals, effect sizes, degrees of freedom and P value noted<br>*Give P values as exact values whenever suitable.*
☒	☐	For Bayesian analysis, information on the choice of priors and Markov chain Monte Carlo settings
☒	☐	For hierarchical and complex designs, identification of the appropriate level for tests and full reporting of outcomes
☐	☒	Estimates of effect sizes (e.g. Cohen's *d*, Pearson's *r*), indicating how they were calculated

Our web collection on statistics for biologists contains articles on many of the points above.

Software and code

Policy information about availability of computer code

Data collection	SoftMax Pro 7.1 (SpectraMax i3 plate reader), NanoSight NS300 software v3.4, Azure cSeries Acquisition software v1.9.5.0606, Azure Sapphire, FACSDiva v9.1, BZ Series Application software v01.01.00.17, Nikon NIS Elements AR 5.21.03, MATLAB 2019b
Data analysis	FlowJo v.10.8.1, GraphPad v.9, ImageJ (Fiji 3), Nikon NIS Elements AR 5.21.03, Microsoft Excel.

For manuscripts utilizing custom algorithms or software that are central to the research but not yet described in published literature, software must be made available to editors and reviewers. We strongly encourage code deposition in a community repository (e.g. GitHub). See the Nature Portfolio guidelines for submitting code & software for further information.

Data

Policy information about availability of data

All manuscripts must include a data availability statement. This statement should provide the following information, where applicable:

- Accession codes, unique identifiers, or web links for publicly available datasets
- A description of any restrictions on data availability
- For clinical datasets or third party data, please ensure that the statement adheres to our policy

Plasmid maps, plasmid descriptions, and plasmids used in each experiment can be found in Supplementary Data 1. Source data are provided with this manuscript. Due to the large size, raw microscopy and flow cytometry data are not included in the source data but are available upon request. Plasmids generated in this study are deposited with Addgene. Bioinformatic analysis was performed using data reported in the SwissProt (https://www.expasy.org/resources/uniprotkb-swiss-prot),

Exocarta (http://www.exocarta.org/), and RaftProt (https://raftprot.org/) databases. Other data are available on reasonable request.
Note: We have included the the statement regarding additional data to be available upon request as a catch all. We believe that all of the data provided in the manuscript, supplemental information, and source data is comprehensive and readers should be able to recapitulate our study and subsequent analysis with the information provided.

Research involving human participants, their data, or biological material

Policy information about studies with human participants or human data. See also policy information about sex, gender (identity/presentation), and sexual orientation and race, ethnicity and racism.

Reporting on sex and gender	N/A
Reporting on race, ethnicity, or other socially relevant groupings	N/A
Population characteristics	N/A
Recruitment	N/A
Ethics oversight	N/A

Note that full information on the approval of the study protocol must also be provided in the manuscript.

Field-specific reporting

Please select the one below that is the best fit for your research. If you are not sure, read the appropriate sections before making your selection.

☒ Life sciences ☐ Behavioural & social sciences ☐ Ecological, evolutionary & environmental sciences

For a reference copy of the document with all sections, see nature.com/documents/nr-reporting-summary-flat.pdf

Life sciences study design

All studies must disclose on these points even when the disclosure is negative.

Sample size	No sample size calculations were performed. The sample sizes for microscopy data (i.e., cells and GPMVs) were n > 15; which is within the standard for the field (see: Levental et al., 2011, PNAS 107 (51), 22050-22054). The sample size for protein gels, vesicle counts, and vesicles sizes was n = 2; where each gel contains ~1e8-1e9 vesicles, and each n is an entirely new vesicle prep (to validate repeatability). Protein gels and blots for n = 2 are often reported (Haakonsen et al. 2024, Nature 626, 874-880). The sample size within each flow cytometry experiment was n = 3, or biological triplicate, which is standard in the field (see Bragdon et al., 2023, Cell 186, 3810-3825).
Data exclusions	No data were excluded from this study.
Replication	In almost all cases, experiments were repeated on separate days in a nearly identical manner to validate experimental results and conclusions. In some cases, a single replicate is reported for simplicity (i.e. flow cytometry dot plots) but the results were similar and repeatable across replicates. Information on replicates can be found in figure captions and methods sections. The western blots in Sup Fig 1 were performed only once; the authors however note that these are standard characterizations used in the extracellular vesicle literature and are repeated for each such publication from our laboratory. The transfected SynTF experiment in Fig 7b (left) was only performed once as described; this was deemed sufficient to support the relevant claims in the main text. In part, this is because these SynTF plasmids have been transfected into reporter cells in other contexts (not reported; such as different well formats or DNA doses) that generated similar results.
Randomization	Samples within an experiment were not arranged randomly, (i.e., within or across well plates), but rather were organized to enable ease of assay completion and/or data presentation. The authors feel randomization in such transfection experiments is not necessary because location of a given well in a well plate is unlikely to strongly affect the results of a given study for these types of experiments.
Blinding	Blinding was not performed in this study. The authors agreed that because the techniques used in this study were quantitative (e.g., flow cytometry), or at least semi-quantitative (protein gels, microscopy), the results were unlikely to be affected by a lack of blinding.

Reporting for specific materials, systems and methods

We require information from authors about some types of materials, experimental systems and methods used in many studies. Here, indicate whether each material, system or method listed is relevant to your study. If you are not sure if a list item applies to your research, read the appropriate section before selecting a response.

Materials & experimental systems

n/a	Involved in the study
☐	☒ Antibodies
☐	☒ Eukaryotic cell lines
☒	☐ Palaeontology and archaeology
☒	☐ Animals and other organisms
☒	☐ Clinical data
☒	☐ Dual use research of concern
☒	☐ Plants

Methods

n/a	Involved in the study
☒	☐ ChIP-seq
☐	☒ Flow cytometry
☒	☐ MRI-based neuroimaging

Antibodies

Antibodies used	See Supplementary Table 7 for complete information on antibodies used to include application, dilution, etc. FLAG (Sigma #F1804), CD9 (sc-13118), CD81 (sc-23962), Alix (Ab117600), Calnexin (Abcam Ab22595), Rabbit (Invitrogen #32460), Mouse (Cell Signaling Technology #7076).
Validation	Per Sigma: "Our standard antibody validation processes include verification for each recommended immunodetection application. Each of the thousands of antibodies in our portfolio are certified through our standard validation process to ensure quality and reproducibility."<br>Per Santa Cruz (sc): "Anti-CD9 Antibody (C-4) is recommended for use in the following applications: WB (Western blotting), IP (Immunoprecipitation), IF (Immunofluorescence), IHC(P) (Immunohistochemistry - Paraffin Embedded), FCM (Flow Cytometry), ELISA (Enzyme-Linked Immunosorbent Assay)...Anti-CD9 Antibody (C-4) has 259 citations in a variety of scientific publications." Also: "Anti-CD81 Antibody (5A6) is recommended for use in the following applications: WB (Western blotting), IP (Immunoprecipitation), IF (Immunofluorescence), FCM (Flow Cytometry)...Anti-CD81 Antibody (5A6) has 109 citations in a variety of scientific publications."<br>Per Abcam: "Antibodies are validated in western blot using lysates from cells or tissues that we have identified to express the protein of interest. Once we have determined the right lysates to use, western blots are run and the band size is checked for the expected molecular weight. We will always run several controls in the same western blot experiment, including positive lysate and negative lysate...When possible, we also include knock-out (KO) cell lines as a true negative control for our western blots...In addition, we run old stock alongside our new stock. If we know the old stock works well, this also acts as a suitable positive control."<br>Per Invitrogen: "Invitrogen antibodies are currently undergoing a rigorous two-part testing approach. Part 1—Target specificity verification. Part 2—Functional application validation"<br>Per Cell Signaling Technology: "We guarantee that our antibodies are fit for purpose by carefully tailoring the combination of validation strategies applied to each product. This means customizing our validation process according to the biological role of the target, while considering the sensitivity requirements of the downstream assay, the availability of appropriate testing models, and the relevance of each method to target investigation."

Eukaryotic cell lines

Policy information about cell lines and Sex and Gender in Research

Cell line source(s)	HEK293FT: Life Technologies / Thermo Fisher (R70007). HEK293FT-LP: Ron Weiss Lab.
Authentication	None.
Mycoplasma contamination	Cell lines tested negative for mycoplasma contamination.
Commonly misidentified lines (See ICLAC register)	None.

Flow Cytometry

Plots

Confirm that:

☒ The axis labels state the marker and fluorochrome used (e.g. CD4-FITC).

☒ The axis scales are clearly visible. Include numbers along axes only for bottom left plot of group (a 'group' is an analysis of identical markers).

☒ All plots are contour plots with outliers or pseudocolor plots.

☒ A numerical value for number of cells or percentage (with statistics) is provided.

Methodology

Sample preparation	HEK293FT and HEK293FT-LP cells were analyzed via flow cytometry. Additional detail is present in the Methods section. Briefly, cells were plated in 24 or 48 well plates and either transfected or incubated with extracellular vesicles. After 2 days, cells were harvested with trypsin and quenched with phenol red-free DMEM. Transfected cells were washed in PBS prior to trypsin treatment. Harvested cells were added to 2 mL flow cytometry buffer, spun down at 150 g x 5 m, and the supernatant

was decanted. Samples were kept light-protected and at 4C until flow cytometry began.

HEK293FT-LP cells were transfected with recombinase and landing pad integration vector and selected for genomic integration using puromycin and blasticidin. Cells were then trypsinized, resuspended in pre-sort medium (DMEM with 10% FBS, 25 mM HEPES, and 100 µg/mL gentamycin), and held on ice until sorting.

Instrument	Analytical flow cytometry was run on a BD LSR Fortessa Special Order Research Product (Robert H. Lurie Cancer Center Flow Cytometry Core). Cell sorting was performed using a BD FACS Aria 4-laser Special Order Research Product (Robert H. Lurie Cancer Center Flow Cytometry Core). Laser and filter configurations are detailed in Methods and Supplementary Information.
Software	FACSDiva software v9.1 was used to collect the data and FlowJo v10.8.1 was used to analyze the data.
Cell population abundance	For cell sorting, approximately 50,000 cells of the final, desired population (see below) were collected and expanded to generate the SynTF reporter line HIE156. For transfected experiments, approximately 3,000-6,000 cells of the desired subpopulation were collected and analyzed. For EV-delivery experiments, approximately 30,000-60,000 cells of the deisred population were collected and analyzed.
Gating strategy	The gating strategy used is detailed in the Supplementary Information. and in the Methods section. Single HEK293FT or HEK293FT-LPs were identified by SSC-A vs FSC-A (cells) and then FSC-H vs FSC-A (singlets). SynTF reporter cells were determined by miRFP720 expression such that approximately < 0.1% of no color, HEK293FT cells were considered miRFP720 +. For transfections, cells transfected with SynTF-encoding DNA were further gating for transfected cells; transfection was determined by the expression of a blue fluorescent protein from a co-transfected plasmid. The "transfected" gate was drawn such that approximately < 0.1% of a cell population transfected with an empty backbone plasmid were considered positive for the blue fluorescent marker. For EV-delivery experiments, activated reporter cells were identified by dsRed-Express2 expression. The "activated" gate was drawn such that approximately < 0.1% of untreated SynTF reporter cells were considered activated.<br><br>HEK293FT-LP cells transfected with recombinase and landing pad integration vector and selected for genomic integration using puromycin were sorted. To gate for cell sorting, single cells were first identified as above. Then, a quadrant gate in FITC (EYFP) and APC-Cy7 (miRFP720) channels was drawn to include on miRFP720+ and EYFP- cells. The quadrant gate was set in the FITC channel such that 99.5% of unmodified HEK293FT cells were considered EYFP-. The quadrant gate was set in the APC-Cy7 channel such that 99.5% of unmodified HEK293FT-LP cells were considered miRFP720-. Only EYFP- and miRFP720+ cells were included in further gating. Of the EYFP-/miRFP720+ cells, the cells in the 88-98th percentile of miRFP720 expression were collected.

☒ Tick this box to confirm that a figure exemplifying the gating strategy is provided in the Supplementary Information.
